# Metabolic Syndrome and Breast Cancer: Prevalence, Treatment Response, and Prognosis

**DOI:** 10.3389/fonc.2021.629666

**Published:** 2021-03-25

**Authors:** Shuwen Dong, Zheng Wang, Kunwei Shen, Xiaosong Chen

**Affiliations:** Department of General Surgery, Comprehensive Breast Health Center, Ruijin Hospital, Shanghai Jiao Tong University School of Medicine, Shanghai, China

**Keywords:** breast cancer, metabolic syndrome, obesity, incidence, treatment response, prognosis

## Abstract

Metabolic syndrome is a type of multifactorial metabolic disease with the presence of at least three factors: obesity, diabetes mellitus, low high-density lipoprotein, hypertriglyceridemia, and hypertension. Recent studies have shown that metabolic syndrome and its related components exert a significant impact on the initiation, progression, treatment response, and prognosis of breast cancer. Metabolic abnormalities not only increase the disease risk and aggravate tumor progression but also lead to unfavorable treatment responses and more treatment side effects. Moreover, biochemical reactions caused by the imbalance of these metabolic components affect both the host general state and organ-specific tumor microenvironment, resulting in increased rates of recurrence and mortality. Therefore, this review discusses the recent advances in the association of metabolic syndrome and breast cancer, providing potential novel therapeutic targets and intervention strategies to improve breast cancer outcome.

## Introduction

Breast cancer is a malignant tumor with the highest incidence in women of all ages in the world and is associated not only with hormones or factors related to reproduction but also with environmental factors in general (1). Epidemiological studies have shown that early menarche, postmenopausal weight gain, a high-fat diet, and long-term use of exogenous estrogen are associated with a high risk of breast cancer (2). Recent studies have also shown that a specific lifestyle characterized by reduced physical activity and fat-rich dietary habits, refined carbohydrates and animal protein, which consequently causes metabolic syndrome (MetS), plays a crucial role in breast cancer initiation (1, 3). Metabolic syndrome, also known as insulin resistance syndrome or syndrome X, is a type of multifactorial metabolic disease. The definition of MetS takes into account the presence of at least three factors, namely, abdominal obesity/high body mass index (BMI), insulin resistance, hypertension, hypertriglyceridemia, and low high-density lipoprotein (HDL) (3–5). The prevalence of obesity has increased rapidly in recent years with the number of overweight/obese people almost doubling since the 1980s, representing one-third of the world’s population, and the proportion may reach 57.8 percent by 2030 (6, 7). In Western countries, the prevalence of MetS in the adult population is between 20% and 25% (8). Notably, the incidence rate increases significantly with age, resulting in the prevalence of people aged over 50 reaching 40-45% (8). Moreover, changes in the balance between insulinotropic and anti-inflammatory cytokines driven by abdominal obesity may lead to insulin resistance, which is a core component of MetS. Asian women are particularly vulnerable to these diseases because they have greater abdominal and visceral fat than white women with similar BMI (9). Previous studies have confirmed that elderly and postmenopausal women are more susceptible to MetS (3). Several cohort studies and meta-analyses have also highlighted the link between MetS and its components and the prevalence, recurrence, and mortality of various cancers, including breast cancer (4, 10, 11).

MetS has become a significant public health problem worldwide, and in-depth research on MetS and breast cancer is increasing. However, there is a paucity of systematic reviews focusing on breast cancer and metabolic syndrome and a comprehensive understanding of this topic. Thus, in this review, we will systematically discuss the latest research advances on MetS and their associations with the incidence, treatment response, prognosis and progression mechanism of breast cancer, which will help provide new therapeutic targets and strategies to improve the prognosis of breast cancer patients.

## Metabolic Syndrome and Breast Cancer Risk

**Figure 1:**
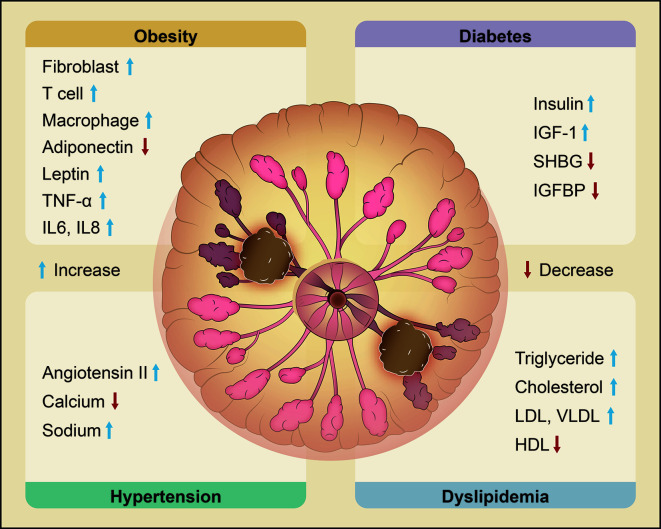
Metabolic syndrome and its molecular changes related to the risk of breast cancer. Obesity, diabetes, hypertension and dyslipidemia are the main components of metabolic syndrome and are all significantly related to the risk of breast cancer. Obesity increases fibroblasts, T cells, macrophages, leptin, TNF-α, IL-6 and IL-8 and decreases adiponectin. Diabetes is characterized by upregulation of insulin and IGF-1 and downregulation of SHBG and IGFBP. Hypertension is associated with increased ANG II and sodium and decreased calcium. Dyslipidemia leads to high levels of TG, TC, LDL, and VLDL and low levels of HDL. Changes in the expression of these key molecules are correlated with an elevated risk of breast cancer. (‘the blue arrow’ means increase and ‘the red arrow’ means decrease).

A population-based study by Russo et al. (12) found that MetS and its components are associated with an increased risk of breast cancer. This connection has been proven in many other studies (13, 14), and it is more pronounced in postmenopausal women regardless of race (odds ratio [OR] = 1.75, 95% confidence interval [CI] 1.37-2.22) (15). With the increase in the number of MetS components, the risk of breast cancer increases for postmenopausal women (*P* = 0.01) (16). Factors related to the risk of breast cancer are discussed in the following four sections (**Figure 1**). **Table 1** summarizes reports about the correlation between metabolic syndrome and its components and the risk of breast cancer with different subtypes.

**Table 1:** Obesity and its measurement indexes with the risk of different breast cancer subtypes.

No.	Author	Patients(N)	Region	Menopausal status	Comparison	Molecular subtype	Measurement	95%CI	*P*	Ref
1	Agresti R. et al.	1779	Italy	Pre-menopausal	BMI≥25 vs. BMI<25	LuminalB(HER2-)	OR=1.30	0.79-2.18	N/A	(13)
						LuminalB(HER2+)	OR=1.78	0.88-3.63	N/A	
						HER2+(non-luminal)	OR=1.89	0.78-4.60	N/A	
						TNBC	OR=3.04	1.43-6.43	N/A	
					WC≥80cm vs. WC<80cm	LuminalB(HER2-)	OR=2.55	1.53-4.24	N/A	
						LuminalB(HER2+)	OR=2.11	1.03-4.35	N/A	
						HER2+(non-luminal)	OR=1.28	0.50-3.27	N/A	
						TNBC	OR=1.03	0.42-2.53	N/A	
				Post-menopausal	BMI≥25 vs. BMI<25	LuminalB(HER2-)	OR=1.51	1.14-2.00	N/A	
						LuminalB(HER2+)	OR=1.20	0.76-1.92	N/A	
						HER2+(non-luminal)	OR=1.43	0.79-2.57	N/A	
						TNBC	OR=1.11	0.65-1.90	N/A	
					WC≥80cm vs. WC<80cm	LuminalB(HER2-)	OR=1.17	0.75-1.81	N/A	
						LuminalB(HER2+)	OR=0.82	0.41-1.63	N/A	
						HER2+(non-luminal)	OR=1.36	0.50-3.69	N/A	
						TNBC	OR=0.89	0.41-1.95	N/A	
2	Chen H. et al.	4557	USA	Both	Type 2 DM vs. non-DM	ER+/HER2+	OR=0.77	0.40-1.48	<0.05	(17)
						TNBC	OR=1.38	1.01-1.89	<0.05	
						H2E	OR=1.38	0.93-2.06	<0.05	
3	Maskarinec G. et al.	681	USA	Both	Type 2 DM in subtypes	ER+/PR+	HR=1.17	1.05-1.29	N/A	(18)
						ER-/PR-	HR=1.03	0.83-1.26	N/A	
						ER+/PR- or ER-/PR+	HR=1.01	0.81-1.28	N/A	
4	Michels K.B. et al.	116488	USA	Both	Type 2 DM vs. non-DM	ER+	HR=1.22	1.01-1.47	N/A	(19)
						ER-	HR=1.13	0.79-1.62	N/A	
5	Millikan R.C. et al.	3446	USA	Pre-menopausal	WHR≥0.84 vs. WHR<0.84	LuminalB	OR=0.90	0.40-1.80	N/A	(20)
						HER2+(non-luminal)	OR=0.90	0.40-2.20	N/A	
						TNBC	OR=1.90	1.00-3.60	N/A	
				Post-menopausal	WHR≥0.84 vs. WHR<0.84	LuminalB	OR=0.50	0.20-0.90	N/A	
						HER2+(non-luminal)	OR=0.50	0.30-1.00	N/A	
						TNBC	OR=1.40	0.70-2.70	N/A	
6	Munsell M.F. et al.	/	/	Pre-menopausal	BMI≥30 vs. BMI<25	ER+/PR+	RR=0.78	0.67-0.92	0.67	(21)
						ER-/PR-	RR=1.06	0.70-1.60	0.004	
				Post-menopausal	BMI≥30 vs. BMI<25	ER+/PR+	RR=1.39	1.14-1.70	0.001	
						ER-/PR-	RR=0.98	0.78-1.22	0.02	
7	Palmer J.R. et al.	1851	USA	Both	Type 2 DM vs. non-DM	ER+	HR=1.02	0.80-1.31	N/A	(22)
						ER-	HR=1.43	1.03-2.00	N/A	
8	Pierobon M. et al.	3845	USA	Pre-menopausal	BMI≥30 vs. BMI<30	TNBC	OR=1.43	1.23-1.65	N/A	(23)
9	Rosner B. et al.	77232	USA	Pre-menopausal	Per 25 lbs weight gain	ER+/PR+	RR=1.13	0.89-1.43	0.32	(24)
						ER+/PR-	RR=2.19	1.33-3.61	0.002	
						ER-/PR-	RR=1.61	1.09-2.38	0.016	
10	Suzuki R. et al.	41594	Japan	Post-menopausal	Per increment of 5kg/m2	ER+/PR+	RR=2.24	1.50-3.34	N/A	(25)
						ER+/PR-	RR=0.63	0.31-1.27	N/A	
						ER-/PR-	RR=0.67	0.38-1.17	N/A	

BMI, Body mass index; ER, Estrogen receptor; HER2, Human epidermal growth factor 2; N/A, Not applicable; OR, Odds ratio; PR, Progesterone receptor; RR, Relative risk; TNBC, Triple negative breast cancer; WC, Waist circumference.

### Obesity and Breast Cancer Risk

Obesity is associated with the risk of postmenopausal breast cancer (26, 27) as well as greater tumor burden and higher histopathological grade (28). BMI is routinely used to measure obesity but lacks information about actual body composition. Waist circumference (WC) and waist-to-hip ratio (WHR) are more commonly adopted for evaluating body fat distribution. Compared with patients with BMI < 25 kg/m^2^, patients with BMI ≥ 30 kg/m^2^ have larger tumors, poorer differentiation, a higher frequency of lymph node invasion, and more advanced disease (29). According to The International Agency for Research on Cancer, ample evidence suggests that increasing weight gain is a risk factor for postmenopausal breast cancer (30). A small case-control study by Schapira et al. (31) found that breast cancer patients had 45% more visceral fat tissue than the control group. In addition, epidemiological studies have shown that WC/WHR increases are related to breast cancer (10, 32, 33). However, some studies have also reported that obesity may have a protective effect on the risk of premenopausal breast cancer (8, 34), which requires further evidence.

Given that excessive estrogen stimulates breast tissue proliferation (35), the increase in estrogen levels in the body caused by obesity is considered to be one of the mechanisms associated with breast cancer because adipose tissue is the main source of estrogen in postmenopausal women (34). Chronic inflammation caused by the imbalance of fat homeostasis due to obesity also promotes the development of tumors. Aromatase is a kind of cytochrome P450 enzyme mainly located in the adipose tissue of breast, abdomen, thigh and buttocks, but it may also be present in tumor tissue. It can catalyze the formation of estrone and estradiol from androstenedione and testosterone (34). Obese adipocytes upregulate the expression of pro-inflammatory factors such as TNF-α and IL-6 through the obesity-inflammation-aromatase axis (36), resulting in enhanced transcription of CYP19 gene encoding aromatase, thereby promoting the production of aromatase. Adipose tissue not only stores adipocytes, it is also an endocrine organ producing biologically active molecules called adipokines. These adipokines bind to specific receptors on the surface of target cells and affect the metabolism of tissues and organs (37). Among the adipokines, leptin increases the risk of disease (38), whereas adiponectin may have a protective effect. Some case-control studies have indicated that low adiponectin levels are associated with increased breast cancer risk and a more aggressive phenotype (39, 40).

For premenopausal women, obesity has a protective effect on hormone receptor-positive breast cancer but increases the risk of estrogen receptor (ER)+/progesterone receptor (PR)- and ER-/PR- breast cancer (24). A meta-analysis conducted by Munsell et al. (21) indicated a summary risk ratio (RR) of 0.78 (95% CI 0.67-0.92) for hormone receptor-positive breast cancer and 1.06 (95% CI 0.70-1.60) for hormone receptor-negative breast cancer in premenopausal women associated with obesity. A positive correlation is noted between obesity and triple-negative breast cancer (TNBC) (23). A multiple logistic regression analysis of 1,779 patients with primary invasive breast cancer in Italy showed that premenopausal women with WC ≥ 80 cm were prone to luminal B breast cancer, including HER2-negative (OR = 2.55, 95% CI 1.53-4.24) and HER2-positive women (OR = 2.11, 95% CI 1.03-4.35), whereas women with BMI ≥ 25 kg/m^2^ (OR = 3.04, 95% CI 1.43-6.43) were significantly related to TNBC compared with other subtypes (13).

For postmenopausal women, obesity is strongly associated with the risk of ER+/PR+ breast cancer but has a weak association with PR- breast cancer (25). Munsell et al. (21) showed in their meta-analysis that obesity was significantly associated with hormone receptor-positive breast cancer in postmenopausal women (RR = 1.39, 95% CI 1.14-1.70), but the RR for hormone receptor-negative breast cancer in postmenopausal women was 0.98 (95% CI 0.78-1.22). A study on the association between BMI and breast cancer subtypes in postmenopausal women in the Mediterranean found that BMI > 25 kg/m^2^ was positively correlated with the risk of luminal breast cancer but not TNBC (41). In a case-control study of a large population conducted by the Carolina Breast Cancer of the United States (20), women with a large WHR regardless of menopausal status (premenopausal OR = 1.9, 95% CI 1.0-3.6; postmenopausal OR = 1.4, 95% CI 0.7-2.7) were more prone to TNBC compared with other breast cancer subtypes.

Therefore, these results indicate that obesity plays a potential role in the prevalence of breast cancer. Obesity may be more likely to increase the risk of hormone receptor-negative breast cancer in premenopausal women and hormone receptor-positive breast cancer in postmenopausal women. In different phenotypes, the risk correlation also varies with measurements of obesity, among which WC and WHR were two important values. Abdominal obesity is correlated with more aggressive molecular types regardless of menopausal status.

### Diabetes Mellitus and Breast Cancer Risk

Diabetes is one of the most frequent chronic diseases in the global population. Insulin resistance is the key factor in the pathogenesis of type 2 diabetes and the most typical and serious phenomenon (42). It is defined as decreased sensitivity to insulin-mediated glucose disposal and inhibition of hepatic glucose production (43) and presents as dysfunction of insulin transduction in glucose uptake and utilization in body skeletal muscles, adipocytes and hepatocytes (44), which leads to hyperglycemia, hyperinsulinemia and various disorders. Insulin resistance is the core part of MetS, and the corresponding increase in fasting blood glucose levels and the effects of hyperinsulinemia in breast cancer are also frequently studied. A meta-analysis (45) showed that compared with the nondiabetic group, the hazard ratio (HR) of the diabetes group was 1.23 (95% CI 1.12-1.34), which indicates that diabetes is a risk factor for breast cancer. In addition, hyperinsulinemia associated with insulin resistance is also correlated with a high risk of breast cancer. Zhu et al. (46) found that the OR for breast cancer associated with the highest quartile versus the lowest quartile of insulin was 1.45 (95% CI 1.20-1.75). Elevated insulin levels can cause high insulin growth factor-1 (IGF-1) bioavailability, leading to the occurrence and proliferation of breast cancer (10). The insulin receptor and IGF-1 receptor are widely expressed in breast cancer cells and promote cell proliferation mainly *via* the insulin receptor substrate (IRS)/phosphatidylinositol 3-kinase (PI3K) and Ras/mitogen-activated protein kinase (MAPK) pathways (8, 39).

Research data have yielded inconsistent reports on the relationship between diabetes mellitus (DM) and the risk of different types of breast cancer. Michels et al. (19) and Maskarinec et al. (18) found that type 2 DM was strongly associated with ER-positive breast cancer (HR for Michels et al. = 1.22, 95% CI 1.01-1.47; HR for Maskarinec et al. = 1.17, 95% CI 1.05-1.29). The main mechanism may be due to crosstalk between estrogen and the insulin/IGF-1 signaling pathway. The activation of ERα is influenced by the insulin/IGF-1 pathway, and estrogen may increase the expression and activity of certain proteins in the pathways that promote signal transduction (47). Palmer et al. (22) observed opposite results. Specifically, a medical history of diabetes was positively correlated with the risk of ER- breast cancer (HR = 1.43, 95% CI 1.03-2.00) rather than ER+ breast cancer. Clinical data indicate that the diabetic state may promote a more aggressive cancer subtype. A retrospective study (17), including 4,557 cases, showed that women diagnosed with type 2 DM have a higher risk of TNBC (OR = 1.38, 95% CI 1.01-1.89) or human epidermal growth factor receptor 2 (HER2)-overexpressing breast cancer (OR = 1.38, 95% CI 0.93-2.06) than patients without a history of diabetes. Several studies also supported that type 2 DM is associated with the risk of TNBC. Regarding antidiabetes treatment, metformin has been indicated to block breast cancer cell cycle progression and selectively induce apoptosis. Previous studies showed that patients treated with metformin had a better breast cancer prognosis (48, 49). Liu et al. (49) reported that metformin has a tumor suppressive effect on breast cancer through a variety of molecular effects, especially on TNBC. They observed that compared with the controls, tumor growth (*P* = 0.0066) and cell proliferation (*P* = 0.0021) in tumor-bearing nude mice treated with metformin were significantly inhibited. Metformin-induced apoptosis, proteolysis of polyadenosine diphosphate-ribose polymerase (PARP) and reduction of epidermal growth factor receptor (EGFR) (a key receptor in TNBC cells) were not observed in phenotypes other than TNBC. Chen et al. (17) found that compared with nondiabetic people, the use of metformin was associated with an increased risk of TNBC (OR = 1.54, 95% CI 1.07-2.22). This may be due to the relatively small sample of women with DM enrolled in the study, and the limited collection of data on history of DM and drug use was only two years before the diagnosis of breast cancer, which limits the evaluation of the impact of long-term use of metformin on the risk of TNBC.

DM and its related hyperinsulinemia and insulin resistance are risk factors for breast cancer. However, the correlation between DM and breast cancer subtypes has inconsistent conclusions among studies and requires further exploration and research. The use of metformin may improve the prognosis of breast cancer patients with diabetes or a prediabetic state, but solid clinical evidence is needed.

### Dyslipidemia and Breast Cancer Risk

Dyslipidemia, including high total triglyceride (TG) levels, high total cholesterol (TC) levels and low serum HDL levels, is also considered to be associated with the occurrence of breast cancer, but the results are inconsistent. A prospective study launched by His et al. (50) showed that TC (HR 1 mmol/L increment = 0.83, 95% CI 0.69-0.99, *P* = 0.04) and HDL (HR 1 mmol/L increment = 0.48, 95% CI 0.28-.83, *P* = 0.009) were inversely associated with the risk of breast cancer. Low serum HDL was independently correlated with an increased risk of breast cancer, especially after menopause (51, 52). However, Kitahara et al. (53) found that TC was positively correlated with breast cancer risk (HR = 1.17, 95% CI 1.03-1.33) in the Korean population. Katzek et al. (54) found that TGs (HR for highest vs. lowest quartile = 0.65, 95% CI 0.46-0.92) were negatively associated with breast cancer risk, but HDL (HR for highest vs. lowest quartile = 1.39, 95% CI 1.01-1.93) was positively associated with breast cancer risk, which differed from previous results. A significant increase in TGs and a decrease in HDL have been observed in TNBC patients (4). Therefore, more clinical data and reliable meta-analyses are needed to confirm the relationship between lipid metabolism and breast cancer risk. A case-control study on Chinese women (33) showed that among all MetS components, the hypertriglyceridemia waist circumference phenotype (HTWC), namely, elevated waist circumference and triglycerides, significantly increased the risk of breast cancer (OR = 1.56, 95% CI 1.02-2.39) regardless of menopausal status. Pelton et al. (55) found that mice fed a high-fat/high-cholesterol diet had significantly higher percentages of tumor cell proliferation and higher microvessel density in preclinical models. Furberg et al. (56) suggested that low serum HDL in overweight and obese women was associated with higher levels of breast mitogens and estrogens; thus, HDL might represent a biological marker of breast cancer risk. In particular, Boyd et al. (57) observed a positive relationship between low HDL levels and atypical hyperplasia of the mammogram.

These results suggest that lipid disorders are a risk factor for breast cancer and may promote tumor cell proliferation and blood vessel formation. However, the relationship between different lipids and the risk of different subtypes of breast cancer still needs to be confirmed through more clinical evidence, so these lipids may be used as biologically reasonable markers to identify and intervene in high-risk individuals. Regarding the possible mechanism by which triglycerides, cholesterol and lipoprotein affect the occurrence and progression of breast cancer, further exploration is needed. Then, new lipid-related strategies can be launched for the prevention and treatment of breast cancer based on the findings.

### Hypertension and Breast Cancer Risk

Regarding hypertension and the risk of breast cancer, the results of studies are not consistent. A cohort study in Finland (58) found no correlation between hypertension and breast cancer (standardized incidence ratio = 0.94, 95% CI 0.84-1.04). Some studies also showed that hypertension was not associated with TNBC (4). A case-control study by Pereira et al. (59) found that women with hypertension (blood pressure ≥ 140/90 mmHg) had a higher risk of breast cancer (OR = 4.18; 95% CI 1.81-9.64), and a significant association was observed in postmenopausal women (OR = 2.84, 95% CI 1.09-7.39). A meta-analysis (60) also showed a significant association between hypertension and increased breast cancer risk (risk ratio [RR] = 1.15, 95% CI 1.08-1.22), especially for postmenopausal women. In contrast, there was no significant correlation with premenopausal women (RR = 0.97, 95% CI 0.84-1.12) or the Asian population (RR = 1.07, 95% CI 0.94-1.22).

Furthermore, Largent et al. (61) additionally studied the impact of the use of antihypertensive drugs on the risk of breast cancer among people with hypertension. Compared to people not receiving antihypertensive treatment, those who used antihypertensive drugs for more than 5 years had a significantly increased risk of invasive breast cancer (RR = 1.18, 95% CI 1.02-1.36), especially ER+ breast cancer (RR = 1.21, 95% CI 1.03-1.42). Among the drugs, people who used diuretics for more than 10 years showed a significant association with the occurrence of breast cancer (RR = 1.16, 95% CI 1.01-1.33), especially ER+ breast cancer (RR = 1.21, 95% CI 1.03-1.42). The possible mechanism is that breast cancer and hypertension have a common pathophysiological pathway mediated by adipose tissue causing chronic inflammation to form metabolic syndrome (62), and hypertension may increase the risk of disease by blocking and changing apoptosis (63). More research is needed to clarify the relationship between hypertension and breast cancer.

## Metabolic Syndrome and Breast Cancer Progression

### Molecular Changes in Patients With Metabolic Syndrome

**Figure 2:**
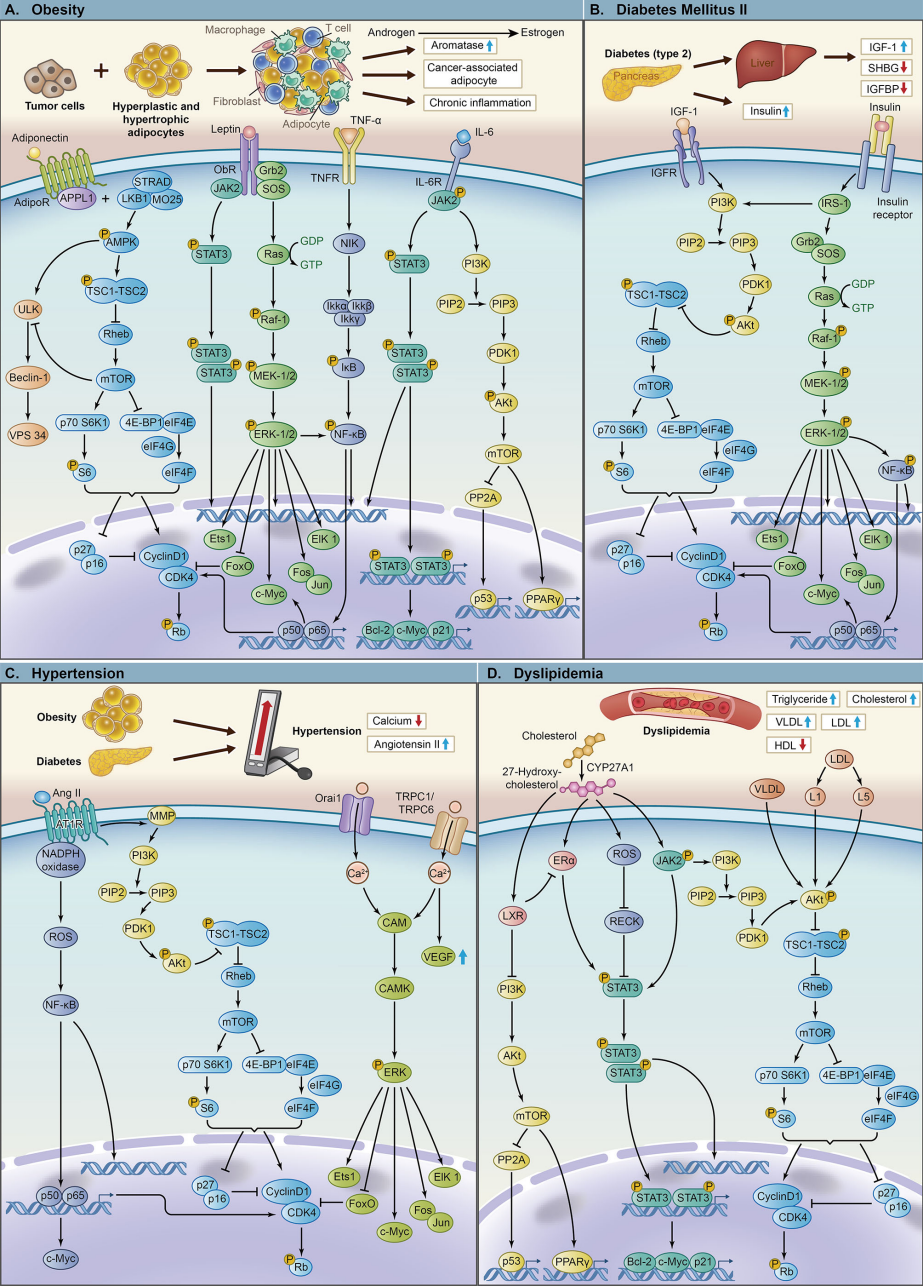
The key pathways and molecular mechanisms of components of metabolic syndrome leading to tumor proliferation, invasion and metastasis. **(A)** The mechanism of obesity and its related key molecules leading to breast cancer with important signaling pathways, including LKB1/AMPK/mTOR, AMPK/ULK, JAK2/STAT3, Ras/Raf/MEK/ERK, NF-κB and PI3K/Akt/mTOR. **(B)** The mechanism of diabetes mellitus type 2 and its related key molecules leading to breast cancer with important signaling pathways, including PI3K/Akt/mTOR, Ras/Raf/MEK/ERK and NF-κB. **(C)** The mechanism of hypertension and its related key molecules leading to breast cancer with important signaling pathways, including NF-κB, PI3K/Akt/mTOR and CAM/CAMK/ERK. **(D)** The mechanism of dyslipidemia and its related key molecules leading to breast cancer with important signaling pathways, including PI3K/Akt/mTOR, JAK2/STAT3 and ERα/STAT3.

As the body’s metabolic state changes, the corresponding molecular level also changes, leading to proliferation, invasion and metastasis of breast cancer *via* various signaling pathways and target genes (**Figure 2**). Under an obese state, the tumor microenvironment changes and produces more fibroblasts and immune cells, such as T cells, macrophages and endothelial cells (64). In the mammary gland, the interaction between obese adipocytes and breast cancer cells leads to the transformation of mammary adipocytes into cancer-associated adipocytes (CAAs) (3), which secrete more leptin and reduce the production of adiponectin. Obese adipose tissue is associated with chronic inflammation, which promotes the production of proinflammatory factors, such as TNF-α, IL-6, and IL-8, and inhibits the secretion of anti-inflammatory factors, such as adiponectin (3, 8). In addition, obese adipose tissue is also associated with increased aromatase activity, which promotes the conversion of androgens to estrogen (65).

Diabetes, especially type 2 diabetes, is often accompanied by insulin resistance, leading to hyperinsulinemia. Diabetes also promotes the production of IGF-1 in the liver (3) and inhibits the secretion of sex hormone-binding globulin (SHBG) (51) and IGF-binding protein (IGFBP) (66).

The renin-angiotensin system (RAS) is the main pathway for regulating blood pressure, and an increase in ANG II (67) leads to hypertension. Serum hypocalcemia, one of the characteristics of hypertension, is also related to the occurrence of tumors.

Dyslipidemia is mainly characterized by high TG and high TC. Elevated cholesterol and increased very-low-density lipoprotein (VLDL) and LDL promote tumor proliferation through different pathways (68).

We reviewed the main findings recent years on the principal molecular actors that are involved in the interactions between MetS and breast cancer biology, including leptin, adiponectin, insulin and IGF-1, Angiotensin II and Calcium and, cholesterol and lipoprotein.

### Leptin

Leptin (**Figure 2A**) is a hormone secreted by adipose cells that acts on the hypothalamus to suppress appetite, increase basal metabolism and inhibit the synthesis of adipocytes by binding to leptin receptors. Leptin is also considered an important biomarker of metabolic syndrome. Elevated levels of leptin are related to obesity, and obese people may exhibit leptin resistance (35, 69). Leptin levels are also proportional to the degree of insulin resistance.

Sieminska et al. (70) found that the leptin level in postmenopausal women was positively correlated with the number of metabolic syndrome components. Leptin is expressed in normal breast tissue, breast cancer cells and solid tumors. Recent research shows that leptin can upregulate aromatase expression by regulating the p53-hypoxia inducible factor 1α/pyruvate kinase M2 axis in breast adipose stromal cells, increasing the risk of breast cancer (71) and promoting the growth of breast tumors. Pan et al. (72) demonstrated that serum leptin levels were related to overall breast cancer risk (standardized mean difference = 0.46, 95% CI 0.31-0.60), especially in Chinese women (standardized mean difference = 0.61, 95% CI 0.44-0.79). Some *in vitro* experiments have shown that leptin can increase the expression of cox-2 and members of the PI3K/Akt pathway in cocultures of leptin and breast cancer cells, such as MCF-7, MDA-MB-231 and BT474, to promote tumor growth (73–75). Leptin has also been found to promote breast cancer invasion and metastasis through the upregulation of procollagen-lysine, 2-oxoglutarate 5-dioxygenase 2 (PLOD2) and IL-18 (76, 77). In *in vivo* experiments, tumor-bearing mouse models showed that leptin increased the volume of tumors and promoted lung metastasis (76). A case-control study by Maccio et al. (78) showed that leptin levels are associated with higher TNM staging and an increased risk of distant metastasis (*P* < 0.05) in postmenopausal breast cancer patients. Regarding potential mechanisms (79), leptin can activate the JAK2/STAT3, PI3K/Akt/mTOR, and extracellular regulated protein kinase (ERK) pathways by binding to its own receptors expressed in tumor cells and stromal components, including immune cells, endothelial cells and tumor-associated fibroblasts. Moreover, it may stimulate the epithelial mesenchymal transition (EMT) (80), which has been confirmed to activate the EMT of breast cancer cells by upregulating pyruvate kinase M2 to activate the PI3K/Akt pathway (81), increasing the activity of matrix metalloproteinases (MMPs), promoting the formation and maintenance of breast cancer stem cell (BCSC) survival, inducing the activation and proliferation of endothelial cells and modulating tumor-immune cell cross talk. Sabol et al. (82) found through molecular experiments that adipose stem cells modified by obesity promoted the secretion of leptin and upregulated and activated the IL-6 and Notch signaling pathways in ER+ breast cancer cells, resulting in ER+ breast cancer radiation resistance. Leptin signaling affects the progression of ER- breast cancer (83). Recent *in vitro* experiments have also found that the expression of leptin receptor and leptin-targeting genes is associated with reduced survival rate of ER- breast cancer and chemotherapy resistance, indicating that the coexpression of leptin receptor and leptin targeting genes can be used as a prognostic indicator of ER-breast cancer patients (84).

Numerous *in vivo* and *in vitro* studies confirmed the role of leptin in recurrence and metastasis. However, relevant clinical evidence is relatively lacking, so more research is needed to investigate leptin as a predictive biomarker and a novel therapeutic target in the clinic.

### Adiponectin

Adiponectin (**Figure 2A**) is a protein synthesized and secreted by white adipocytes. Obese patients have lower levels of adiponectin compared with normal patients. In contrast to leptin, the concentration of adiponectin decreases as the number of metabolic syndrome components increases (85–88). Adiponectin activates various signaling pathways (AMPK, PI3K/Akt, MAPK, PPAR-α, STAT3 and NF-κB) (39) and exerts various cellular functions by binding to two receptors, adiponectin receptor 1 (AdipoR1) and 2 (AdipoR2). Adiponectin improves insulin sensitivity *in vivo* by activating PPAR-α (89). Based on the role of adiponectin in the abovementioned signaling pathways, the short peptide ADP355 based on adiponectin can exert antiproliferative effects in breast cancer cells through the STAT3 and ERK1/2 signaling pathways (39, 90). Adiponectin also increases insulin sensitivity in muscles and liver through the AMPK pathway and improve insulin resistance (91) as an endogenous insulin sensitizer. Adiponectin deficiency causes downregulation of the activity of PTEN and activation of the PI3K/Akt signaling pathway, thereby promoting breast tumors (92). In addition, adiponectin controls inflammation, inhibits angiogenesis (93), reduces proliferation, and promotes apoptosis. It inhibits the expression of adhesion molecules in endothelial cells, suppresses the growth of macrophage precursors and downregulates TNF-α and IL-6/-8 to control inflammation (94). Adiponectin potentially inhibits angiogenesis through the AMPK pathway to enhance nitric oxide production, activate endothelial nitric oxide synthase (95), and suppress the expression of pro-angiogenesis factors, such as vascular endothelial growth factor (VEGF) and IL-8. *In vitro* experiments further demonstrated that in MCF-7 and MDA-MB-231 cell lines, the proliferation of endothelial cells expressing adiponectin receptors is inhibited by adiponectin, thereby reducing VEGF expression and inhibiting metastasis, invasion, and angiogenesis (96). Moreover, adiponectin may inhibit TNF-α-induced activation of the NF-κB pathway to promote apoptosis.

Adiponectin is negatively related to the risk of breast cancer. Many studies have shown that low adiponectin levels are associated with the risk of breast cancer and the progression of more aggressive subtypes (40), which is more common in ER-/PR- breast cancer. *In vivo* experiments showed that in animals receiving ER- MDA-MB-231 cells, the tumor volume was significantly reduced after adiponectin pretreatment (97). Moreover, Oh et al. (98) found that adiponectin may protect against recurrence in ER-/PR- breast cancer patients. Although adiponectin exhibits antiproliferative effects on cell growth in both ER+ and ER- cell lines, the main mechanisms may be different. For ER+ breast cancer, a low adiponectin concentration can amplify ER signaling to increase the proliferation of ER+ breast cancer cells, leading to the progression of breast tumors (40, 99, 100). Low adiponectin levels may mediate the proliferation of ER- breast cancer cells by regulating genes involved in the cell cycle (such as p53, Bax, Bcl-2, c-myc and Cyclin D1) (101), which indicates that adiponectin can be used as adjuvant therapy for ER- breast cancer, and its local and systemic application can reduce tumor size and inhibit tumor metastasis.

The possible mechanism by which adiponectin exerts antitumor growth and proautophagy effects is the LKB1-AMPK-mTOR/ULK pathway (102). *In vitro* experiments have shown that adiponectin increases the expression of the tumor suppressor gene LKB1 in breast cancer cells and subsequently reduces tumor adhesion, migration and invasion through the AMPK-p70S6 kinase (S6K) axis (103). Adiponectin binds to the C-terminus of AdipoR, and its N-terminus binds to the PTB domain of signal adaptor protein (APPL1), affecting LKB1 expression. In obese patients, adiponectin production is reduced such that binding to AdipoR is inhibited. The binding of the domain of APPL1 to LKB1 is suppressed, so AMPK and ULK expression is consequently inhibited (104). Some studies have summarized that the *in vitro* data of adiponectin’s antitumor proliferation effect in breast cancer are mainly limited to full-length adiponectin (fAd) (105, 106), one of the forms of adiponectin. The other form of adiponectin, globular adiponectin (gAd), may have the opposite effect. It can activate AMPK through its high-affinity receptor AdipoR1 acting on aggressive tumor cells, and AMPK upregulates autophagy by activating ULK1 to enhance tumor metastasis (106). Mauro et al. further found that gAd inhibits the growth of ER+ breast cancer but promotes ER+ breast cancer proliferation (107).

Based on adiponectin-mediated cytotoxic autophagy, we can reasonably hypothesize that adiponectin usage represents a new adjuvant therapy strategy for obese breast cancer patients and that combining adiponectin with chemotherapeutic drugs may therefore reduce the dose of chemotherapeutic drugs (108). Studies have concluded (109) that leptin and adiponectin receptors seem to be the most promising molecular targets for the treatment of metabolic syndrome-related cancers. In the future adjuvant therapy of breast cancer, for patients with metabolic syndrome, it may be possible to improve the efficacy by adjusting the circulating levels of the above molecules to obtain a better prognosis.

### Insulin and IGF-1

Insulin (**Figure 2B**) is an anabolic hormone. It has mitogenic, antiapoptotic and angiogenic effects, which are partially related to cancer progression and mortality. Hyperinsulinemia can also affect the prognosis of breast cancer mainly through the following mechanisms. Insulin itself promotes the synthesis of DNA, RNA, and ATP, induces mitosis and angiogenesis, and inhibits apoptosis. High insulin levels induce aromatase activity (110), increase the chance of conversion of androgen to estrogen, and promote mitosis. Moreover, high insulin levels also reduce the production of SHBG synthesized by the liver (111) and increase the proportion of circulating estradiol, leading to the growth of breast cancer. *In vitro* experiments proved that insulin induces EMT, invasion and migration of tumor cells (112). In a chronic hyperinsulinemic state, the ER is activated to promote tumor growth by regulating the cell cycle, apoptosis factors, and nutrient metabolism (113). This mechanism provides a basis for using metformin to treat ER+ breast cancer patients with diabetes. In addition, hyperinsulinemia mediates the production of IGF-1 (**Figure 2B**), inhibits the production of IGFBP in the liver, increases the bioavailability of IGF-1 and stimulates further tumor growth through excessive activation of the PI3K-Akt-mTOR pathway and Ras-MAPK pathway (114).

MetS patients with insulin resistance have high circulating insulin levels. Insulin binds to the insulin receptor on the cell membrane and activates IRS1 and subsequently activates the Ras/Raf/MEK/ERK pathway to regulate cell proliferation and differentiation. Insulin also stimulates the production of IGF-1 in the liver and downregulates the secretion of IGFBP1 and 2 in the liver, leading to increased bioavailability of circulating IGF-1 (66, 115). IGF-1 receptor (IGF-1R) is a transmembrane receptor tyrosine kinase that is similar in structure to the insulin receptor. It is often overexpressed in breast cancer cells and regulates cell proliferation, survival, differentiation, and transformation (116–119). IGF-1 combines with IGFR, which activates PI3K and thereby activates Akt. Activated Akt further induces the mTOR pathway, leading to cell proliferation and protein synthesis in tumor cells.

Therefore, improving insulin resistance through the above pathways can inhibit tumor growth and proliferation directly or indirectly, and it may also relieve other metabolic abnormalities that can improve the curative effect of adjuvant therapy, leading to better disease prognosis.

### Angiotensin II and Calcium

RAS and calcium (**Figure 2C**) are hypertension-related molecules. Studies have reported the relationship and possible mechanism between these molecules and breast cancer. RAS is an important physiological mechanism of hypertension, and studies have shown that it also plays an important role in the progression of breast cancer. It has been observed in some *in vitro* and *in vivo* experiments that all components of the RAS are overexpressed in breast cancer cells (67). Among them, angiotensin II (Ang II) (**Figure 2C**) is a well-known hypertension hormone that binds to angiotensin II type 1 receptor (AT1R) and angiotensin II type 1 receptor (AT2R). Ang II-AT1R is related to breast cancer proliferation, and its mechanism has been extensively studied. After Ang II binds to AT1R, AT1R interacts with nicotinamide adenine dinucleotide phosphate (NADPH) oxidase to activate AT1R and promotes the infiltration of macrophages and the production of VEGF and reactive oxygen species (ROS). ROS further promote members of the downstream NF-κB pathway to bind to target genes and mediate cell transcription. AT1R also promotes the secretion of matrix metalloproteinases and then activates the PI3K/Akt/mTOR pathway to promote tumor growth and inhibit cell apoptosis.

Calcium ions are also an important part of the pathogenesis of hypertension. Studies have found that calcium ion channels are associated with tumor proliferation, angiogenesis, apoptosis and metastasis. In hypertensive patients, calcium ions flow through calcium channels, resulting in intracellular high calcium and extracellular low calcium. A meta-analysis by Wulaningsih et al. (120) found that serum calcium had a protective effect on breast cancer (RR 0.80, 95% CI 0.66-0.97). Deliot et al. (121) showed that calcium ions flood into cells through the Orai1 channel (**Figure 2C**), phosphorylating ERK *via* the calcium/calcium-dependent calmodulin (CAM)/calmodulin kinase II (CAMK) pathway, which regulates cell proliferation. In addition, Orai1 levels in TNBC were increased compared with that in non-TNBC (122). Orai1 is a 33kDa protein with four transmembrane domains and its functional channel is a hexamer composed of 6 Orai1 subunits (123). Orai1 forms a highly selective calcium ion channel in the plasma membrane to mediate the transmission of calcium ions (124). The antiproliferative and antimigratory effects of Orai1 silencing were observed in the MDA-MB-231 basal breast cancer cell line. This finding reveals that the Orai1 channel may represent a therapeutic target for TNBC. Calcium can also stimulate breast cancer cell proliferation and activate VEGF through the (transient receptor potential-canonical) TRPC6/TRPC1 channel (**Figure 2C**). The use of calcium channel blockers may inhibit the growth of breast cancer tumors. The use of calcium-channel blockers for 10 or more years was associated with a greater than twofold increase in the risk of ductal breast cancer (OR 2.4, 95% CI 1.2-4.9) and lobular breast cancer (OR 2.6, 95% CI 1.3-5.3) according to Li et al. (125).

### Cholesterol and Lipoprotein

High cholesterol (**Figure 2D**) is an important feature of dyslipidemia in obese patients and one of the side effects of adjuvant therapy in patients. It has a certain impact on the pathophysiology and progression of breast cancer. Nelson et al. (126) found that 27-hydroxycholesterol (27HC), a primary metabolite of cholesterol and an ER and liver X receptor (LXR) ligand, might play an important role in the progression of breast cancer. Cholesterol is converted into 27HC by cytochrome P450 oxidase. Then, 27HC activates ER and LXR to promote breast cancer cell growth and metastasis. Nelson et al. further confirmed that in mouse models of breast cancer, 27HC significantly reduced tumor latency (*P* < 0.05) and accelerated tumor growth (*P* < 0.05). Increases in markers, such as cell proliferation, angiogenesis, and macrophage infiltration, were also observed in mice treated with 27HC. In higher grade tumors, increased expression of cytochrome P450 oxidase was observed (OR 6.7, 95% CI 1.7-27, *P* = 0.0007), indicating that cancer cells could increase 27HC levels through autocrine signaling. In addition, 27HC activates the STAT3 pathway by reactive oxygen species (ROS) methylation and activation of JAK and extracellular ERα. LXR suppresses the PI3K/Akt pathway to inhibit cell proliferation and downregulate ER expression (127), and ER enhances STAT3 activation (128).

Epidemiologically, abnormal lipoprotein levels (**Figure 2D**) have also been shown to be significantly associated with breast cancer. Data from Lu et al. (68) provide a new mechanism by which lipoproteinemia promotes tumor development. They found that the VLDL, L1 and L5 subfractions of LDL **(** **Figure 2D** **)** activated Akt to promote cell migration by Ser473 phosphorylation. VLDL, L1, and L5 also increase mesenchymal markers, such as vimentin and β-catenin, to induce EMT and enhance angiogenic factors in breast cancer to promote angiogenesis. Therefore, reducing circulating cholesterol or inhibiting its conversion to 27HC and abnormal lipoprotein levels may represent new strategies for the prevention and/or treatment of breast cancer. However, the effect of lipid composition on the growth of breast cancer cells and its mechanism still require more research.

## Metabolic Syndrome and Treatment Effect on Breast Cancer

### Metabolic Syndrome and Adjuvant Therapy Efficacy

Healy et al. (129) reported that MetS is associated with more aggressive tumor biology, such as later tumor stage (*P* = 0.022) and lymph node invasion (*P* = 0.028). Compared with breast cancer patients who do not have metabolic abnormalities, those with MetS and its components show worse treatment responses to various therapies.

Regarding chemotherapy, a study by Stebbing et al. (130) on the relationship between MetS and response to chemotherapy for breast cancer found that among patients with metabolic syndrome, the proportion of progression, stability and response to treatment after chemotherapy ranged from 61.1% to 42.9% to 33.3% (*P* for trend = 0.03). This finding indicated that the MetS-mediated risk of nonresponse to chemotherapy was higher and that treatment efficacy was worse. Litton et al. (131) found that overweight (BMI = 25-29 kg/m^2^) and obese (BMI ≥ 30 kg/m^2^) groups exhibited more difficulties than normal or underweight (BMI < 25) groups in achieving pathologic complete response (pCR) (OR = 0.67, 95% CI 0.45-0.99) with neoadjuvant chemotherapy. This unsatisfactory treatment response may be due to underdose in obese patients (132). In the guidelines issued by the American Society of Clinical Oncology, full-dose chemotherapy based on body weight was recommended to obese patients because their worsening survival may be related to the insufficient dose of cytotoxic therapy (41, 133). However, considering safety and therapeutic benefits, overweight and obese patients worldwide are still receiving reduced doses of chemotherapy (23, 134).

DM can also affect the efficacy of chemotherapy. The elevated concentration of IGF-1 under hyperglycemia can specifically inhibit cell death induced by anticancer drugs in MCF-7 cells, which suggests its involvement in the mechanism of drug resistance. Zeng et al. (135) showed that hyperglycemia-induced resistance to chemotherapy was only observed in ER+ breast cancer cells, indicating that antiestrogen may promote the effectiveness of chemotherapy in such patients.

For endocrine therapy, Ewertz et al. (29) followed up on 18,967 Danish women with BMI information who received early breast cancer treatment found that after one decade, in contrast to the patients with BMI < 25 kg/m^2^, endocrine therapy in patients with BMI ≥ 30 kg/m^2^ had a worse response. Compared to normal weight women, the disease-free survival (DFS) rate (HR = 1.78, 95% CI 1.12-2.83) and overall survival (OS) rate (HR = 2.28, 95% CI 1.16-4.51) are worse in obese women receiving tamoxifen (TAM) combined with aromatase inhibitors (AIs) (136). In this regard, they further studied the role of fulvestrant and TAM in chemotherapy resistance. Fulvestrant is a selective estrogen receptor degrader that can not only competitively bind to ER, but also induce ER degradation and down-regulate ER levels. Fulvestrant blocked hyperglycemia-mediated chemotherapeutic resistance, but TAM did not (135). Diabetes is another major factor affecting endocrine therapy, which may lead to a worse treatment response (8, 137). For DM patients, IGF-1 receptor or IRS-1 overexpression increases the resistance of breast cancer cells to antiestrogen therapy (138).

Trastuzumab is a widely used targeted medicine. It is a monoclonal antibody against HER2, affecting the transmission of growth signals through specific binding to the HER2 receptor, and at the same time kill tumor cells through antibody-dependent cell-mediated cytotoxicity pathway. For targeted therapy, obese adipocytes secrete more IGF-1, thereby increasing drug resistance, such as trastuzumab resistance (139), but the underlying mechanism remains unclear. Lee et al. (140) demonstrated in their research that in HER2+ breast cancer patients receiving trastuzumab treatment, DM was a significant unfavorable prognostic factor for DFS (*P* = 0.006) and OS (*P* = 0.017), which was consistent with previous reports (141).

For radiation therapy, in the study of Sabol et al. (82), when breast cancer cells were cocultured with obesity-altered adipose stem cells, breast cancer cell apoptosis decreased and survival increased after radiotherapy. Research has suggested that obesity-altered adipose stem cells upregulate IL-6 and activate the Notch signaling pathway to induce radiation tolerance. Fang et al. (142) found that higher BMI was associated with worse quality of life for breast cancer patients before, during, and after radiotherapy even after adjusting for other factors. DM patients also showed worse response and radiation tolerance through elevation of IGF-1R and IRS-1 (47).

### Adjuvant Therapy and Metabolic Changes

A published observational study (143) showed that after patients completed corresponding chemotherapy, metabolic syndrome and its components were significant risk factors (*P* < 0.01). Among them, the fasting blood glucose level changed by 20.3% and the triglyceride level changed by 18.4%, the deterioration of which were more significant. Several studies have demonstrated that breast cancer patients who have received chemotherapy are more likely to gain weight than those who have not (144–146). Fredslund et al. (147) also observed consistent results. Changes were evident in body fat (*P* = 0.01), triglycerides (*P* = 0.03), WC (*P* = 0.008), glucose (*P* = 0.02) and diastolic blood pressure (*P* = 0.04) of premenopausal women, whereas changes in WC (*P* = 0.03), HDL (*P* = 0.05) and glucose (*P* = 0.02) were observed in postmenopausal women. These findings suggested that chemotherapy may have greater adverse effects in premenopausal breast cancer patients because this group may be induced to undergo either transient or permanent early menopause that promotes the rapid decline of estradiol levels in the body, leading to weight gain and a rapid and continuous increase in fat mainly in the abdomen, which causes a series of changes in metabolism-related components. Such weight gain may also increase the risk of chemotherapy-related diabetes. Glucocorticoids used in combination with chemotherapeutic drugs or chemotherapeutic drugs themselves, such as platinum and cyclophosphamide, can cause abnormal glucose metabolism through direct or indirect mechanisms and worsen the pre-existing DM state in susceptible individuals (148). Among the three breast cancer treatments, including radiotherapy, chemotherapy, and endocrine therapy, evaluated in the analysis conducted by Bordelea et al. (149), only chemotherapy (*P* = 0.03) was related to new-onset diabetes.

Studies have shown that endocrine therapy, such as estrogen inhibitors, may also lead to worse metabolic status. TAM is a commonly used antiestrogen drug that can cause dyslipidemia, a well-known side effect with a worsening HDL level and circulating TG level, in women with breast cancer (150). However, TAM was reported to lower circulating TC and (low-density lipoprotein) LDL (151). Fatty liver (*P =* 0.000) and visceral adipose tissue (*P =* 0.000) are also significant side effects of receiving TAM (152).

Furthermore, the use of TAM can lead to increased fasting blood glucose levels and insulin resistance even when administered in low doses. A population-based cohort study launched by Sun et al. (153) demonstrated that TAM users showed a significantly increased risk of DM compared with non-TAM users among breast cancer patients (adjusted HR = 1.31, 95% CI 1.19-1.45). TAM also leads to a decrease in insulin sensitivity. Johansson et al. (150) observed a 7-fold decrease in insulin sensitivity among breast cancer patients using TAM (OR = 0.15, 95% CI 0.03-0.88) compared with nonusers. The underlying mechanism of the link between TAM and diabetes is still unclear. A possible explanation is that estrogen maintains steady blood glucose, and TAM may affect the interaction between estrogen and insulin by inhibiting estrogen. Additionally, hypertriglyceridemia and fatty liver caused by TAM are features of insulin resistance and glucose intolerance (153).

AIs, including letrozole, anastrozole and exemestane, are another important hormone therapy. Similar to TAM, abnormal lipid metabolism is one of the most significant side effects of AI, but the specific impact is different. Compared with TAM, AI may not change TG levels but increase TC and LDL-C levels. Among AIs, anastrozole and letrozole increase TC and LDL-C levels, whereas exemestane decreases TC, LDL-C, TG and HDL-C levels (151, 154). An early *in vitro* study showed that 17-hydroxy exemestane, a metabolite of exemestane, may elicit androgenic effects by binding to the androgen receptor. Bell et al. (154) hypothesized that this may be a potential mechanism by which exemestane could reduce HDL cholesterol. However, less is known about nonsteroidal aromatase inhibitors, such as anastrozole and letrozole. Hong et al. (155) showed in their cohort study that the incidence rate of fatty liver was increased in the TAM group than in the AI group (*P* = 0.021). The reason may be that TAMs increase circulating TG levels and promote insulin resistance, thereby promoting susceptibility to fatty liver. These findings also suggested that TAMs have direct liver toxicity, but more evidence is needed to confirm this hypothesis (155).

In addition, hypertension is a characteristic adverse event of bevacizumab therapy. The incidence was 17.9% in a clinical study (156). The possible mechanism is that bevacizumab inhibits vascular endothelial growth factor (VEGF), which enhances endothelial nitric oxide synthase activity. These effects lead to decreased production of vasodilator nitric oxide, which causes hypertension (157).

Thus, an interaction exists between adjuvant therapy and human body metabolic status. Patients with unstable metabolic status will receive unfavorable results from adjuvant therapy. Conversely, adjuvant therapy will also cause deterioration of the human body’s metabolic status.

### Obesity and Cardiotoxicity of Trastuzumab and Anthracyclines

Compared with chemotherapy alone, the combination of targeted medicine and chemotherapy has clinical advantages in improving the DFS and OS rates of breast cancer patients (158–160). Anthracyclines and trastuzumab are widely used in the treatment of breast cancer patients, and both can induce acute or chronic dose-dependent cardiotoxicity, especially acute cardiac insufficiency, which is the most serious side effect. This condition is characterized by reduced left ventricular ejection fraction (LVEF), which is typically asymptomatic or associated with heart failure (161). A meta-analysis of 8,754 breast cancer patients treated with anthracyclines, sequential anthracyclines (anthracyclines followed by trastuzumab), and trastuzumab launched by Guenancia et al. (162) showed that in an unadjusted analysis, overweight plus obesity was obviously related to the risk of cardiotoxicity of the above therapies. The OR was 1.38 (95% CI 1.06-1.80) for overweight plus obesity, 1.47 (95% CI 0.95-2.28) for obese individuals and 1.15 (95% CI 0.83-1.58) for overweight individuals. Subgroup analysis showed that the risk of cardiotoxicity gradually increased with increasing BMI (*P* = 0.05). For different administrations, the rate of cardiotoxicity of obese patients treated with anthracyclines only was 20% (95% CI 5%-43%), and obese patients treated with trastuzumab with or without anthracyclines had a 16% (95% CI 10%-24%) rate of cardiotoxicity.

Gunaldi et al. (163) found an association between postmenopausal women (*P* = 0.01) and cardiotoxicity caused by trastuzumab. Obesity (*P* = 0.0001) and hypertension (*P* 0.002) were related to lower LVEF in patients, whereas diabetes (*P* = 0.766) was not statistically significant. However, a retrospective study (164) observed a correlation between a history of diabetes and trastuzumab-related cardiotoxicity (*P* = 0.01). Therefore, evaluation of heart function or appropriate dose reduction is needed for obese breast cancer patients before treatment with anthracycline or trastuzumab. During and after treatment, LVEF should also be evaluated regularly to prevent heart insufficiency and heart failure. The relationship between people with DM and the incidence of cardiotoxicity caused by trastuzumab requires further exploration.

Obese patients or mice fed a high-fat diet were more sensitive to the cardiotoxicity caused by anthracyclines (165, 166). The mechanism may be due to downregulation of cardiac peroxisome proliferator-activated receptor-α, decreased mitochondrial adenosine monophosphate (AMP)-α2 protein kinase and a reduction in cardiac adenosine triphosphate (ATP) levels after doxorubicin administration according to Mitra et al. (166). Therefore, high-fat diet-induced obese rats are highly sensitized to anthracycline-induced cardiotoxicity by downregulating cardiac mitochondrial ATP generation, increasing oxidative stress and downregulating the Janus kinase (JAK)/signal transducers and activators of transcription 3 (STAT3) pathway. Additionally, Maruyama et al. (167) found that adiponectin-knockout mice showed aggravated left ventricular systolic dysfunction after injection of doxorubicin, whereas exogenous adiponectin improved this condition in wild-type and adiponectin-knockout mice. These results indicate that downregulation of adiponectin levels partly affects adverse cardiac reactions, suggesting that adiponectin may be used as a therapy to prevent cardiotoxicity caused by anthracyclines in obese breast cancer patients. More evidence is needed to confirm the underlying mechanism of trastuzumab-induced cardiotoxicity in obese patients.

## Metabolic Syndrome and Prognosis of Breast Cancer

### Recurrence

Current evidence suggests that metabolic syndrome and its components are associated with an increased risk of breast cancer recurrence (3, 168). A retrospective study conducted by Ewertz et al. (29) assessed the events of locoregional recurrence and distant metastasis among 53,816 women with early-stage breast cancer up to 10 years after diagnosis and showed that obesity may have no effect on the risk of local recurrence (*P* > 0.05) but had a significant association with distant metastasis 5 to 10 years after diagnosis. The HR of distant metastasis in patients with a BMI of 25 to 29 kg/m^2^ was 1.42 (95% CI 1.17-1.73, *P* < 0.001). The HR in patients with a BMI of 30 kg/m^2^ or more was 1.46 (95% CI 1.11-1.92, *P* = 0.007). In particular, adiponectin is an important adipocytokine, and its expression is reduced in obese people. Oh et al. (98) found that compared to the highest quartile of serum adiponectin levels, the lowest quartile showed a 2.82-fold increased risk.

Goodwin et al. (169) demonstrated that, regardless of menopausal status, fasting plasma insulin levels are associated with high tumor grade, axillary lymph node involvement, and risk of recurrence. Patients in the highest quartile of insulin level had an increased risk of distant recurrence (HR 2.0, 95% CI 1.2-3.3, *P* = 0.007). However, in different molecular types of breast cancer, the correlation between insulin resistance and recurrence risk shows different results (98). In contrast with ER-/PR- patients, a negative association is noted between insulin resistance and tumor recurrence in ER+/PR+ people, which is consistent with serum insulin levels. Moreover, ER+/PR+ patients with hyperglycemia exhibited a lower risk of recurrence (HR = 0.48, 95% CI 0.26-0.89, *P* = 0.020) (**Table 2**).

**Table 2:** The effect of metabolic syndrome and its components on recurrence and survival of breast cancer.

No.	Author	Pts(N)	Region	Study design	Molecular subtype	Comparison	Outcome	HR	95%CI	*P*	Ref
1	Buono G. et al.	717	Italy	prospective observational study	All	1-2MetS components vs. 0MetS component	OS	4.90	1.47-16.35	0.01	(3)
							BCSS	6.07	1.41-26.21	0.02	
						3-5MetS components vs. 0MetS component	OS	12.20	3.49-43.01	<0.0001	
							BCSS	15.97	3.49-73.16	<0.0001	
2	Cho W. K. et al.	5668	Korea	retrospective cohort study	All	BMI≥25 vs. BMI<25	OS	1.356	1.038-1.773	0.03	(146)
							DFS	1.248	1.038-1.502	0.076	
						Non-hyperlipidemia vs. Hyperlipidemia	OS	3.085	1.836-5.183	<0.001	
							DFS	1.447	1.080-1.937	0.013	
3	Emaus A. et al.	1364	Norway	retrospective cohort study	All	BMI≥30 vs. BMI=18.5-25	OS	1.47	1.08-1.99	N/A	(170)
						highest tertile of cholesterol vs. lowest	OS	1.29	1.01-1.64	N/A	
						highest tertile of blood pressure vs. lowest	OS	1.41	1.09-1.83	N/A	
4	Ewertz M. et al.	18967	Denmark	retrospective cohort study	All	BMI≥30 vs. BMI<25	BCSS	1.38	1.11-1.71	0.003	(29)
5	Minicozzi P. et al.	1607	Italy	retrospective cohort study	ER/PR+	High glucose(>94.0mg/dl) vs. reference (84.1-94.0mg/dl)	BCSS	5.49	1.56-19.31	N/A	(171)
					ER-/PR-		BCSS	0.77	0.15-4.17	N/A	
6	Oh S. W. et al.	747	Korea	retrospective cohort study	ER/PR+	Hyperglycemia vs. non-hyperglycemia	Recurrence	0.48	0.26-0.89	0.02	(98)
					ER-/PR-	Serum adiponectin	Recurrence	N/A	N/A	0.009	

BCSS, Breast cancer specific survival; BMI, Body mass index; DFS, Disease free survival; ER, Estrogen receptor; HR, Hazard ratio; MetS, Metabolism Syndrome; N/A, Not applicable; OS, Overall Survival; PR, Progesterone receptor; Pts, patients.

The impact of dyslipidemia on the prognosis of breast cancer has demonstrated conflicting results. In general, breast cancer patients with high levels of TC, low density lipoprotein-cholesterol (LDL-C) and TG and low levels of HDL-cholesterol (HDL-C) show poor prognosis (170, 172). However, a retrospective study by Jung et al. (173) observed opposite results. In the study, compared to high levels of LDL-C (quartile IV) and TGs (quartile IV), lower levels of LDL-C (quartile III) (HR 1.88, 95% CI 1.09-3.27, *P* = 0.02) and abnormally low levels of TGs (quartile I) (HR 1.88, 95% CI 1.07-3.29, *P* = 0.03) showed obviously higher risks of recurrence. Ozdemir et al. (174) also found that the risk of recurrence was increased in patients with normocholesterolemia compared with patients with hypercholesterolemia (*P* = 0.001). However, Bahl et al. (172) found no statistically significant relationship between lipids and breast cancer outcome other than a trend toward a risk of recurrence with higher TC (HR 1.62 for the fourth to first quartile, 97.5% CI 0.98-2.69, *P* = 0.03) in multivariate analysis. Therefore, the potential role of lipids in the recurrence of breast cancer should be further studied.

Regarding hypertension, Braithwaite et al. (175) showed that hypertension was another independent predictor of prognosis in breast cancer. Interestingly, hypertension was associated with recurrence among African Americans (HR 1.60, 95% CI 1.07-2.40) but not their white counterparts. However, the reason was not clear, and more studies are needed to explain the distinction. The association among other ethnic groups requires more solid results.

The above findings indicate that MetS and its composition can be regarded as predictors of recurrence. Among them, evaluating adiponectin and insulin concentrations can help determine the prognosis in ER-/PR- patients, and corresponding interventions can be implemented to improve the prognosis and reduce the risk of recurrence. In addition, more research is needed to prove the impact of serum lipid levels and hypertension on the recurrence of breast cancer and the relationship between MetS and its components and breast cancer subtypes. Therefore, it could provide new and specific prognostic detection methods to monitor and prevent recurrence in breast cancer patients.

### Mortality

For patients with early-stage breast cancer, abnormal metabolic status will bring unsatisfactory outcomes. MetS is widely thought to be associated with a risk of mortality for breast cancer patients, and the risk sharply increases as the number of its components increases. Compared with individuals without any metabolic syndrome-related components, patients with 1-2 components have a 5-fold higher risk of death (HR = 4.90, 95% CI 1.47-16.35, *P* = 0.01) and a 6-fold higher risk of breast cancer-specific death (HR = 6.07, 95% CI 1.41-26.21, *P* = 0.02) (**Table 2**); patients with 3-5 components have a 12-fold higher risk of death (HR = 12.2, 95% CI 3.49-43.01, *P* < 0.0001). In addition, the risk of breast cancer-specific death is 16-fold higher (HR = 15.97, 95% CI 3.49-73.16, *P* < 0.0001) (3).

Epidemiologists show that the mortality rate of breast cancer among Asians is the lowest in the world, but it has rapidly increased in recent years due to the sharp increase in obesity and consequent metabolic disorders (176, 177). It was proven that obese women show a worse survival rate regardless of menopausal status. A study from the United States in 2009 found that after diagnosis with breast cancer, every gained 5 kg of body weight could increase breast cancer-specific mortality by 13% (66). Cho et al. (146) found that in all patients, BMI ≥ 25 kg/m^2^ was an unfavorable factor for OS (*P* = 0.030). In a Danish retrospective study of 18,967 early breast cancer patients (29), patients with BMI ≥ 30 kg/m^2^ at the time of diagnosis had a 38% increase in breast cancer mortality compared with patients with a BMI < 25 kg/m^2^ (HR = 1.38, 95% CI 1.11-1.71). In addition, studies have also found that obesity was significantly associated with adverse outcomes in women with ER+ tumors, and obesity had an impact on ER- or HER2+ tumors (40, 178). The possible mechanisms may be due to obesity-related molecules, such as leptin, adiponectin, TNF-α, and IL-6, and their subsequent signal pathways. These findings also suggested that obese patients often have tumors detected later along with more aggressive tumor biological characteristics; therefore, fewer treatment opportunities with poorer therapeutic effects are available, which leads to an increased risk of death.

Diabetes is also associated with high breast cancer mortality, and a meta-analysis showed a significantly higher all-cause mortality risk (HR 1.49, 95% CI 1.35-1.65) of patients with breast cancer and diabetes (179). Another meta-analysis also indicated that HR was 1.51 (95% CI 1.34-1.70) for OS and 1.28 (95% CI 1.09-1.50) for DFS in breast cancer patients with diabetes compared to those without (180). However, its effects differ among subtypes. ER+/PR+ women with hyperglycemia are more likely to die of breast cancer (HR = 5.49, 95% CI 1.56-19.31), whereas ER-/PR- patients show no significant association (171). The underlying reasons may be that patients with diabetes receive less aggressive treatment because they are vulnerable to related comorbidities and may have a greater risk of chemotherapy-related toxicity. Additionally, diabetes can directly affect breast cancer by altering related molecules, such as insulin, IGF-1 and inflammatory markers. Bozcuk et al. (181) and Pasanisi et al. (182) found that fasting serum insulin levels are an independent predictor of OS in breast cancer patients, which may be related to the high expression of insulin receptors in breast cancer tissues.

Notably, in the study of Cho et al. (146), the absence of hyperlipidemia is an unfavorable prognostic factor for DFS (HR 1.447, 95% CI 1.080-1.937, *P* = 0.013) and OS (HR 3.085, 95% CI 1.836-5.183, *P* < 0.001) in breast cancer patients, which is perhaps related to the use of statins in patients with hyperlipidemia. A Norwegian research team found (170) that breast cancer patients with high total cholesterol levels (HR for OS is 1.29, 95% CI 1.01-1.64, *P* = 0.03) had a high risk of overall mortality. This finding may be attributed to the fact that cholesterol contributes to the progression and metastasis of tumors *via* several pathways, such as Akt and EGFR. Fan et al. (14) found that in the TNBC group, patients with low HDL showed worse relapse-free survival (RFS) (HR 3.266, 95% CI 2.087-5.112, *P* < 0.0001) and OS (HR 3.071, 95% CI 1.732-5.445, *P* < 0.0001). The possible mechanism is that HDL negatively correlates with angiotensin (ANG) II, which is positively associated with VEGF pathways in TNBC cells.

Therefore, MetS and its components play a profound role in the survival of breast cancer patients. In individuals, positive associations are observed between obesity as well as diabetes and mortality. Hyperlipidemia shows a protective effect on the mortality of breast cancer. The exact association between MetS and its related components and different phenotypes and underlying mechanisms remain unclear and require further study.

### Anti-MetS and Prognosis

Healy et al. (129) reported that MetS is associated with more aggressive tumor biology, such as later tumor stage (*P* = 0.022) and lymph node invasion (*P* = 0.028). Interventions, including medications, such as metformin and statins, low-calorie and low-fat diets and appropriate exercise to improve metabolic disorders, are needed to reduce the risk of comorbidities in breast cancer patients. The measures to treat MetS may consequently have a positive impact on the prognosis of patients to a certain extent.

Metformin is widely used in the treatment of hyperglycemia. Jiralersong et al. (183) showed that during neoadjuvant chemotherapy for breast cancer, patients with diabetes who received metformin had a higher pathologic complete response (pCR) rate (24%, 95% CI 13%-34%) compared with those who did not (8%, 95% CI 2.3%-14%). These researchers additionally indicated that metformin use during neoadjuvant chemotherapy was an independent predictor of pCR (OR 2.95, 95% CI 1.07-8.17, *P* = 0.04). Given the differences in insulin usage among the enrolled population, they further analyzed the effect of insulin usage on pCR. In the metformin group, the rate of pCR was not different for insulin use vs. no insulin use (27% vs. 23%, *P* = 0.75). Metformin plays a role in inhibiting the proliferation, invasion and angiogenesis of tumor cells by reversing hyperinsulinemia (183) and improving insulin resistance (34). Some *in vitro* studies have shown that the antitumor effect of metformin *via* epidermal growth factor receptor (EGFR)-mediated pathways (49) is most prominent in TNBC cell lines. In addition, metformin may target the immune microenvironment of tumors to inhibit tumor proliferation (184).

It was reported that breast cancer patients without hyperlipidemia had worse DFS (HR 1.447, 95% CI 1.080-1.937, *P* = 0.013) and OS (HR 3.085, 95% CI 1.836-5.183, *P* < 0.001) (146), which may be due to the use of statins. The retrospective analysis of Li et al. (185) found that long-term use of statins (> 5 years) was associated with improvements in OS (HR = 0.38, 95% CI 0.17-0.85, *P* < 0.018) and DFS (HR = 0.15, 95% CI 0.05-0.48, *P* < 0.001), even after adjusting for metabolic comorbidities. There was no significant difference in OS between patients taking statins for less than 5 years and those who did not take statins. A randomized phase III trial (186) conducted by the International Breast Organization on 8,010 patients with early postmenopausal hormone receptor-positive invasive cancer indicated that compared with patients who did not use cholesterol-lowering drugs, patients who received cholesterol-lowering drugs before endocrine therapy had better DFS (HR = 0.82, 95% CI 0.68-0.99). *In vitro*, statins inhibit the proliferation of the breast cancer cell line MCF-7, which may be due to the blockade of hydroxy methyl glutaryl coenzyme A (HMG-CoA) reductase (187). Ghosh-Choudhury et al. (188) found that simvastatin significantly inhibits the phosphorylation of Akt kinase in MDA-MB-231 breast cancer cells and further inhibits the mammalian target of rapamycin (mTOR) pathway. Statins also induce apoptosis in a variety of cancer cell lines, including colon, prostate, and breast cancer cells (185). Additionally, statins are well tolerated, and their drug interactions are limited. Among different statin types, lipophilic drugs show direct inhibition of breast cancer cell growth *in vitro* and *in vivo*, whereas, hydrophilic drugs such as pravastatin, have no effect (187, 189, 190).

For a long time, aerobic exercise has been widely believed to be effective in improving the abnormal metabolic state of the body, such as reducing fasting blood sugar, HDL, TGs, and WC. Resistance exercise can induce changes in insulin sensitivity by maintaining and/or increasing lean body mass, increasing glucose storage, reducing circulating glucose levels, and promoting a decrease in the amount of insulin required by obese people. A prospective study of 1,490 women with breast cancer conducted by Women’s Healthy Eating and Living reported that the equivalent of walking 30 minutes a day for 6 days a week plus eating at least 5 servings of fruit and vegetables a week significantly benefited survival (HR = 0.56, 95% CI 0.31-0.98). However, in the analysis of subtypes, this lifestyle intervention can only reduce the mortality of ER+ tumors (*P* < 0.05).

Adhering to the Mediterranean diet is another option to reduce the occurrence of metabolic syndrome, preventing the prevalence of breast cancer and improving its prognosis. Some cohort studies have observed a corresponding risk reduction (191–193). The main characteristics of the Mediterranean diet are extensive consumption of fruits, vegetables, unrefined grains, legumes, fish, cereals, nuts, olive oil, and moderate drinking of wine during the main meal (194). Shifting to a Mediterranean diet can improve the imbalance of body metabolism (195) and prevent intractable diseases related to insulin resistance, such as obesity and breast cancer (196). A systemic review published in 2008 confirmed that the Mediterranean diet has a significant negative correlation with the risk of postmenopausal ER- breast cancer (197). Reducing inflammation may be a possible mechanism for the anticancer effect of the Mediterranean diet (1).

Therefore, medications for metabolic disorders and specific lifestyles may be effective strategies to improve the outcome of breast cancer patients, especially those with MetS, after diagnosis to obtain a better prognosis. These research conclusions may provide us with innovative treatments.

## Conclusion

Metabolic syndrome and its components have been widely considered to be correlated with the initiation and progression of breast cancer, which is due to obesity and its related adipokines, insulin and IGFs, abnormal serum lipids and lipoproteins and the molecules leading to hypertension. These molecular changes partly exert a profound influence on the tumor and its microenvironment. Metabolic syndrome is significantly associated with an increased risk, worse treatment response, invasive progression and poor prognosis of breast cancer. Notably, we systematically reviewed the mechanisms and pathways of the highlighted molecules affecting disease progression and summarized several potentially novel treatment targets. In the future, new treatment strategies can be prospectively performed based on the above findings to improve prognosis and improve quality of life for breast cancer patients.

## Author Contributions

SD, ZW, and XC designed this study. SD performed the search and analysis. SD and ZW wrote the manuscript. XC and KS helped to revise the manuscript. All authors contributed to the article and approved the submitted version.

## Funding

This study was supported by the National Natural Science Foundation of China (81772797, 82072897, and 82002773), Shanghai Municipal Education Commission—Gaofeng Clinical Medicine Grant Support (20172007), Ruijin Hospital, Shanghai Jiao Tong University School of Medicine-Guangci Excellent Youth Training Program (GCQN-2017-A18), and Ruijin Youth NSFC Cultivation Fund (2019QNPY01046).

## Conflict of Interest

The authors declare that the research was conducted in the absence of any commercial or financial relationships that could be construed as a potential conflict of interest.

## Glossary

**Table:**

27HC	27-hydroxycholesterol
AdipoR	adiponectin receptor
AI	aromatase inhibitor
AMP	adenosine monophosphate
AMPK	adenosine monophosphate kinase
ANG	angiotensin
APPL1	adaptor protein containing the pleckstrin homology domain, phosphotyrosine-binding domain, and leucine zipper motif 1
AT1R	angiotensin II type 1 receptor
AT2R	angiotensin II type 2 receptor
ATP	adenosine triphosphate
BMI	body mass index
BCSC	breast cancer stem cell
CAA	cancer-associated adipocytes
CAM	calmodulin
CAMK	calmodulin kinase
CI	confidence interval
DFS	disease-free survival
DM	diabetes mellitus
DNA	deoxyribonucleic acid
EGFR	epidermal growth factor receptor
EMT	epithelial mesenchymal transition
ER	estrogen receptor
ERK	extracellular regulated protein kinases
fAd	full-length adiponectin
gAd	globular adiponectin
HDL	high-density lipoprotein
HER2	human epidermal growth factor receptor 2
HMG-CoA	hydroxy methyl glutaryl coenzyme A
HTWC	hypertriglyceridemia waist circumference
IGF-1	insulin growth factor-1
IGFBP	insulin growth factor binding protein
IL	interleukin
IRS	insulin receptor substrate
JAK	Janus kinase
LDL	low density lipoprotein
LKB1	liver kinase B1
LVEF	left ventricular ejection fraction
LXR	liver x receptor
MAPK	mitogen-activated protein kinase
MEK	mitogen-activated protein kinase
MetS	metabolic syndrome
MMP	matrix metalloproteinases
mTOR	mammalian target of rapamycin
NADPH	nicotinamide adenine dinucleotide phosphate
NF-kB	nuclear factor kappa-B
OR	odds ratio
OS	overall survival
PARP	poly adenosine diphosphate-ribose polymerase
pCR	pathologic complete response
PI3K	phosphatidylinositol 3-kinase
PLOD2	procollagen-lysine, 2-oxoglutarate 5-dioxygenase 2
PPAR	peroxisome proliferators-activated receptors
PR	progesterone receptor
PTB	phosphotyrosine binding
RAS	renin-angiotensin system
RFS	relapse-free survival
RNA	ribonucleic acid
ROS	reactive oxygen species
RR	risk ratio
S6K	p70S6 kinase
Ser	serine
SHBG	sex hormone binding globulin
STAT3	signal transducers and activators of transcription 3
TAM	tamoxifen
TC	total cholesterol
TG	total triglyceride
TNBC	triple negative breast cancer
TNF	tumor necrosis factor
TRPC	transient receptor potential-canonical
ULK	unc-51-likekinase
VEGF	vascular endothelial growth factor
VLDL	very-low-density-lipoprotein
WC	waist circumference
WHR	waist-to-hip ratio

## References

[1] Iacoviello L Bonaccio M Gaetano G Donati MB. Epidemiology of breast cancer, a paradigm of the “common soil” hypothesis. Semin Cancer Biol (2020). 10.1016/j.semcancer.2020.02.010 32087245

[2] Stoll BA. Timing of weight gain and breast cancer risk. Ann Oncol (1995) 6:245–8. 10.1093/oxfordjournals.annonc.a059153 7612489

[3] Buono G Crispo A Giuliano M De Angelis C Schettini F Forestieri V. Metabolic syndrome and early stage breast cancer outcome: results from a prospective observational study. Breast Cancer Res Treat (2020) 182:401–9. 10.1007/s10549-020-05701-7 PMC7297840 32500397

[4] Maiti B Kundranda MN Spiro TP Daw HA. The association of metabolic syndrome with triple-negative breast cancer. Breast Cancer Res Treat (2010) 121:479–83. 10.1007/s10549-009-0591-y 19851862

[5] Kabat GC Kim M Chlebowski RT Khandekar J Ko MG McTiernan A. A longitudinal study of the metabolic syndrome and risk of postmenopausal breast cancer. Cancer Epidemiol Biomarkers Prev (2009) 18:2046–53. 10.1158/1055-9965.EPI-09-0235 PMC6204126 19567502

[6] Kelly T Yang W Chen CS Reynolds K He J. Global burden of obesity in 2005 and projections to 2030. Int J Obes (Lond) (2008) 32:1431–7. 10.1038/ijo.2008.102 18607383

[7] Bray F Ferlay J Soerjomataram I Siegel RL Torre LA Jemal A. Global cancer statistics 2018: GLOBOCAN estimates of incidence and mortality worldwide for 36 cancers in 185 countries. CA Cancer J Clin (2018) 68:394–424. 10.3322/caac.21492 30207593

[8] Hauner D Hauner H. Metabolic syndrome and breast cancer: is there a link? Breast Care (Basel) (2014) 9:277–81. 10.1159/000365951 PMC4209278 25404888

[9] Lim U Ernst T Buchthal SD Latch M Albright CL Wilkens LR. Asian women have greater abdominal and visceral adiposity than Caucasian women with similar body mass index. Nutr Diabetes (2011) 1:1–8. 10.1038/nutd.2011.2 PMC3302135 23449381

[10] Uzunlulu M Telci Caklili O Oguz A. Association between Metabolic Syndrome and Cancer. Ann Nutr Metab (2016) 68:173–9. 10.1159/000443743 26895247

[11] Esposito K Chiodini P Colao A Lenzi A Giugliano D. Metabolic syndrome and risk of cancer: a systematic review and meta-analysis. Diabetes Care (2012) 35:2402–11. 10.2337/dc12-0336 PMC3476894 23093685

[12] Russo A Autelitano M Bisanti L. Metabolic syndrome and cancer risk. Eur J Cancer (2008) 44:293–7. 10.1016/j.ejca.2007.11.005 18055193

[13] Agresti R Meneghini E Baili P Minicozzi P Turco A Cavallo I. Association of adiposity, dysmetabolisms, and inflammation with aggressive breast cancer subtypes: a cross-sectional study. Breast Cancer Res Treat (2016) 157:179–89. 10.1007/s10549-016-3802-3 27117160

[14] Fan Y Ding X Wang J Ma F Yuan P Li Q. Decreased serum HDL at initial diagnosis correlates with worse outcomes for triple-negative breast cancer but not non-TNBCs. Int J Biol Markers (2015) 30:200–7. 10.5301/jbm.5000143 25953090

[15] Rosato V Bosetti C Talamini R Levi F Montella M Giacosa A. Metabolic syndrome and the risk of breast cancer in postmenopausal women. Ann Oncol (2011) 22:2687–92. 10.1093/annonc/mdr025 21415236

[16] Wang M Cheng N Zheng S Wang D Hu X Ren X. Metabolic syndrome and the risk of breast cancer among postmenopausal women in North-West China. Climacteric (2015) 18:852–8. 10.3109/13697137.2015.1071346 26507498

[17] Chen H Cook LS Tang MTC Hill DA Li CI. Relationship between Diabetes and Diabetes Medications and Risk of Different Molecular Subtypes of Breast Cancer. Cancer Epidemiol Biomarkers Prev (2019) 28:1802–8. 10.1158/1055-9965.EPI-19-0291 PMC6825551 31395589

[18] Maskarinec G Jacobs S Park SY Haiman CA Setiawan VW Wilkens LR. Type II Diabetes, Obesity, and Breast Cancer Risk: The Multiethnic Cohort. Cancer Epidemiol Biomarkers Prev (2017) 26:854–61. 10.1158/1055-9965.EPI-16-0789 PMC5457323 28087607

[19] Michels KB SC Hu FB Rosner BA Hankinson SE Colditz GA Manson JE. Type 2 diabetes and subsequent incidence of breast cancer in the Nurses’ Health Study. Diabetes Care (2003) 26:1752–8. 10.2337/diacare.26.6.1752 12766105

[20] Millikan RC Newman B Tse CK Moorman PG Conway K Dressler LG. Epidemiology of basal-like breast cancer. Breast Cancer Res Treat (2008) 109:123–39. 10.1007/s10549-007-9632-6 PMC2443103 17578664

[21] Munsell MF Sprague BL Berry DA Chisholm G Trentham-Dietz A. Body mass index and breast cancer risk according to postmenopausal estrogen-progestin use and hormone receptor status. Epidemiol Rev (2014) 36:114–36. 10.1093/epirev/mxt010 PMC3873844 24375928

[22] Palmer JR Castro-Webb N Bertrand K Bethea TN Denis GV. Type II Diabetes and Incidence of Estrogen Receptor Negative Breast Cancer in African American Women. Cancer Res (2017) 77:6462–9. 10.1158/0008-5472.CAN-17-1903 PMC5726548 29141994

[23] Pierobon M Frankenfeld CL. Obesity as a risk factor for triple-negative breast cancers: a systematic review and meta-analysis. Breast Cancer Res Treat (2013) 137:307–14. 10.1007/s10549-012-2339-3 23179600

[24] Rosner B Eliassen AH Toriola AT Hankinson SE Willett WC Natarajan L. Short-term weight gain and breast cancer risk by hormone receptor classification among pre- and postmenopausal women. Breast Cancer Res Treat (2015) 150:643–53. 10.1007/s10549-015-3344-0 PMC4383816 25796612

[25] Suzuki R Iwasaki M Inoue M Sasazuki S Sawada N Yamaji T. Body weight at age 20 years, subsequent weight change and breast cancer risk defined by estrogen and progesterone receptor status–the Japan public health center-based prospective study. Int J Cancer (2011) 129:1214–24. 10.1002/ijc.25744 21064092

[26] White AJ Nichols HB Bradshaw PT Sandler DP. Overall and central adiposity and breast cancer risk in the Sister Study. Cancer (2015) 121:3700–8. 10.1002/cncr.29552 PMC4592412 26193782

[27] Neuhouser ML Aragaki AK Prentice RL Manson JE Chlebowski R Carty CL. Overweight, Obesity, and Postmenopausal Invasive Breast Cancer Risk: A Secondary Analysis of the Women’s Health Initiative Randomized Clinical Trials. JAMA Oncol (2015) 1:611–21. 10.1001/jamaoncol.2015.1546 PMC5070941 26182172

[28] Feigelson HS Patel AV Teras LR Gansler T Thun MJ Calle EE. Adult weight gain and histopathologic characteristics of breast cancer among postmenopausal women. Cancer (2006) 107:12–21. 10.1002/cncr.21965 16718671

[29] Ewertz M Jensen MB Gunnarsdottir KA Hojris I Jakobsen EH Nielsen D. Effect of obesity on prognosis after early-stage breast cancer. J Clin Oncol (2011) 29:25–31. 10.1200/JCO.2010.29.7614 21115856

[30] Calle EE RC Walker-Thurmond K Thun MJ. Overweight, Obesity, and Mortality from Cancer in a Prospectively Studied Cohort of U.S. Adults. N Engl J Med (2003) 348:1625–38. 10.1056/NEJMoa021423 12711737

[31] Schapira D Clark R Wolff P Jarrett A Kumar N Aziz N. Visceral obesity and breast cancer risk. Cancer (1994) 74:632–9. 10.1002/1097-0142(19940715)74:2<632::aid-cncr2820740215>3.0.co;2-t 8033042

[32] Davis AA Kaklamani VG. Metabolic syndrome and triple-negative breast cancer: a new paradigm. Int J Breast Cancer (2012) 2012:809291. 10.1155/2012/809291 22295251 PMC3262602

[33] Xiang Y Zhou W Duan X Fan Z Wang S Liu S. Metabolic Syndrome, and Particularly the Hypertriglyceridemic-Waist Phenotype, Increases Breast Cancer Risk, and Adiponectin Is a Potential Mechanism: A Case-Control Study in Chinese Women. Front Endocrinol (Lausanne) (2019) 10:905. 10.3389/fendo.2019.00905 32038481 PMC6990117

[34] Wysocki P Wierusz-Wysocka B. Obesity, hyperinsulinemia and breast cancer: novel targets and a novel role for metformin. Expert Rev Mol Diagn (2010) 10:509–19. 10.1586/erm.10.22 20465505

[35] Lorincz AM Sukumar S. Molecular links between obesity and breast cancer. Endocr Relat Cancer (2006) 13:279–92. 10.1677/erc.1.00729 16728564

[36] Howe LR Subbaramaiah K Hudis CA Dannenberg AJ. Molecular pathways: adipose inflammation as a mediator of obesity-associated cancer. Clin Cancer Res (2013) 19:6074–83. 10.1158/1078-0432.CCR-12-2603 PMC3891839 23958744

[37] Zorena K Jachimowicz-Duda O Slezak D Robakowska M Mrugacz M. Adipokines and Obesity. Potential Link to Metabolic Disorders and Chronic Complications. Int J Mol Sci (2020) 21:3570. 10.3390/ijms21103570 PMC7278967 32443588

[38] Christodoulatos GS Spyrou N Kadillari J Psallida S Dalamaga M. The Role of Adipokines in Breast Cancer: Current Evidence and Perspectives. Curr Obes Rep (2019) 8:413–33. 10.1007/s13679-019-00364-y 31637624

[39] Mauro L Naimo GD Ricchio E Panno ML Ando S. Cross-Talk between Adiponectin and IGF-IR in Breast Cancer. Front Oncol (2015) 5:157. 10.3389/fonc.2015.00157 26236690 PMC4502352

[40] Naimo GD Gelsomino L Catalano S Mauro L Ando S. Interfering Role of ERalpha on Adiponectin Action in Breast Cancer. Front Endocrinol (Lausanne) (2020) 11:66. 10.3389/fendo.2020.00066 32132979 PMC7041409

[41] Crispo A Montella M Buono G Grimaldi M D’Aiuto M Capasso I. Body weight and risk of molecular breast cancer subtypes among postmenopausal Mediterranean women. Curr Res Transl Med (2016) 64:15–20. 10.1016/j.retram.2016.01.004 27140595

[42] Marunaka Y. The Proposal of Molecular Mechanisms of Weak Organic Acids Intake-Induced Improvement of Insulin Resistance in Diabetes Mellitus via Elevation of Interstitial Fluid pH. Int J Mol Sci (2018) 19:3244. 10.3390/ijms19103244 PMC6214001 30347717

[43] Gutch M Kumar S Razi SM Gupta KK Gupta A. Assessment of insulin sensitivity/resistance. Indian J Endocrinol Metab (1999) 19:160–4. 10.4103/2230-8210.146874 PMC4287763 25593845

[44] Morgan B Chai S Albiston A. GLUT4 associated proteins as therapeutic targets for diabetes. Chem Biol Interact (2018) 280:33–44. 10.2174/187221411794351914 22074575

[45] Bruijn KM Arends LR Hansen BE Leeflang S Ruiter R Eijck CH. Systematic review and meta-analysis of the association between diabetes mellitus and incidence and mortality in breast and colorectal cancer. Br J Surg (2013 100:1421–9. 10.1002/bjs.9229 24037561

[46] Zhu Y Wang T Wu J Huang O Zhu L He J. Biomarkers of Insulin and the Insulin-Like Growth Factor Axis in Relation to Breast Cancer Risk in Chinese Women. Onco Targets Ther (2020) 13:8027–36. 10.2147/OTT.S258357 PMC7429223 32848423

[47] Lanzino M Morelli C Garofalo C Panno ML Mauro L Andò S. Interaction between estrogen receptor alpha and insulin/IGF signaling in breast cancer. Curr Cancer Drug Targets (2008) 8:597–610. 10.2174/156800908786241104 18991569

[48] Evans JMM Morris AD. Research Pointers: Metformin and reduced risk of cancer in diabetic patients. BMJ Br Med J (2005) 330:1304. 10.1136/bmj.38415.708634.F7 15849206 PMC558205

[49] Liu B Fan Z Edgerton SM Deng XS Alimova IN Lind SE. Metformin induces unique biological and molecular responses in triple negative breast cancer cells. Cell Cycle (2009) 8:2031–40. 10.4161/cc.8.13.8814 19440038

[50] His M Zelek L Deschasaux M Pouchieu C Kesse-Guyot E Hercberg S. Prospective associations between serum biomarkers of lipid metabolism and overall, breast and prostate cancer risk. Eur J Epidemiol (2014) 29:119–32. 10.1007/s10654-014-9884-5 24519551

[51] Vona-Davis L Howard-McNatt M Rose DP. Adiposity, type 2 diabetes and the metabolic syndrome in breast cancer. Obes Rev (2007) 8:395–408. 10.1111/j.1467-789X.2007.00396.x 17716297

[52] Michalaki V Koutroulis G Koutroulis G Syrigos K Piperi C Kalofoutis A. Evaluation of serum lipids and high-density lipoprotein subfractions (HDL2, HDL3) in postmenopausal patients with breast cancer. Mol Cell Biochem (2005) 268:19–24. 10.1007/s11010-005-2993-4 15724433

[53] Kitahara C Berrington de González A Freedman N Huxley R Mok Y Jee S. Total cholesterol and cancer risk in a large prospective study in Korea. J Clin Oncol: Off J Am Soc Clin Oncol (2011) 29:1592–8. 10.1200/jco.2010.31.5200 PMC3082977 21422422

[54] Katzke V Sookthai D Johnson T Kühn T Kaaks R. Blood lipids and lipoproteins in relation to incidence and mortality risks for CVD and cancer in the prospective EPIC-Heidelberg cohort. BMC Med (2017) 15:218. 10.1186/s12916-017-0976-4 29254484 PMC5735858

[55] Pelton K Coticchia CM Curatolo AS Schaffner CP Zurakowski D Solomon KR. Hypercholesterolemia induces angiogenesis and accelerates growth of breast tumors in vivo. Am J Pathol (2014) 184:2099–110. 10.1016/j.ajpath.2014.03.006 PMC4076468 24952430

[56] Furberg AS Jasienka G Bjurstam N Torjesen PA Emaus A Lipson SF. Metabolic and hormonal profiles: HDL cholesterol as a plausible biomarker of breast cancer risk. The Norwegian EBBA Study. Cancer Epidemiol Biomarkers Prev (2005) 14:33–40. 15668473

[57] Boyd N McGuire V Fishell E Kuriov V Lockwood G Tritchler D. Plasma lipids in premenopausal women with mammographic dysplasia. Br J Cancer (1989) 59:766–71. 10.1038/bjc.1989.160 PMC2247212 2736211

[58] Lindgren AM Nissinen AM Tuomilehto JO Pukkala E. Cancer pattern among hypertensive patients in North Karelia, Finland. J Hum Hypertension (2005) 19:373–9. 10.1038/sj.jhh.1001834 15703772

[59] Pereira A Garmendia M Alvarado M Albala C. Hypertension and the risk of breast cancer in Chilean women: a case-control study. Asian Pac J Cancer Prev (2012) 13:5829–34. 10.7314/apjcp.2012.13.11.5829 23317264

[60] Han H Guo W Shi W Yu Y Zhang Y Ye X. Hypertension and breast cancer risk: a systematic review and meta-analysis. Sci Rep (2017) 7:44877. 10.1038/srep44877 28317900 PMC5357949

[61] Largent J Bernstein L Horn-Ross P Marshall S Neuhausen S Reynolds P. Hypertension, antihypertensive medication use, and breast cancer risk in the California Teachers Study cohort. Cancer Causes Control (2010) 21:1615–24. 10.1007/s10552-010-9590-x PMC2941047 20526803

[62] Li JJ Fang CH Hui RT. Is hypertension an inflammatory disease? Med Hypotheses (2005) 64:236–40. 10.1016/j.mehy.2004.06.017 15607546

[63] Hamet P. Cancer and hypertension: a potential for crosstalk? J Hypertens (1997) 15:1573–7. 10.1097/00004872-199715120-00058 9488208

[64] Donohoe CL Lysaght J O’Sullivan J Reynolds JV. Emerging Concepts Linking Obesity with the Hallmarks of Cancer. Trends Endocrinol Metab (2017) 28:46–62. 10.1016/j.tem.2016.08.004 27633129

[65] Wang X Simpson ER Brown KA. Aromatase overexpression in dysfunctional adipose tissue links obesity to postmenopausal breast cancer. J Steroid Biochem Mol Biol (2015) 153:35–44. 10.1016/j.jsbmb.2015.07.008 26209254

[66] Chen Y Wen YY Li ZR Luo DL Zhang XH. The molecular mechanisms between metabolic syndrome and breast cancer. Biochem Biophys Res Commun (2016) 471:391–5. 10.1016/j.bbrc.2016.02.034 26891869

[67] Rasha F Ramalingam L Gollahon L Rahman RL Rahman SM Menikdiwela K. Mechanisms linking the renin-angiotensin system, obesity, and breast cancer. Endocr Relat Cancer (2019) 26:653–72. 10.1530/ERC-19-0314 31525726

[68] Lu CW Lo YH Chen CH Lin CY Tsai CH Chen PJ. VLDL and LDL, but not HDL, promote breast cancer cell proliferation, metastasis and angiogenesis. Cancer Lett (2017) 388:130–8. 10.1016/j.canlet.2016.11.033 27940127

[69] Izquierdo AG Crujeiras AB Casanueva FF Carreira MC. Leptin, Obesity, and Leptin Resistance: Where Are We 25 Years Later? Nutrients (2019) 11:2704. 10.3390/nu11112704 PMC6893721 31717265

[70] Siemińska L Wojciechowska C Foltyn W Kajdaniuk D Zemczak A. The relation of serum adiponectin and leptin levels to metabolic syndrome in women before and after the menopause. Endokrynol Pol (2006) 57:15–22. 16575758

[71] Zahid H Subbaramaiah K Iyengar N Zhou X Chen I Bhardwaj P. Leptin regulation of the p53-HIF1α/PKM2-aromatase axis in breast adipose stromal cells: a novel mechanism for the obesity-breast cancer link. Int J Obes (Lond) (2018) 42:711–20. 10.1038/ijo.2017.273 PMC5936686 29104286

[72] Pan H Deng L Cui J Shi L Yang Y Luo J. Association between serum leptin levels and breast cancer risk: An updated systematic review and meta-analysis. Medicine (2018) 97:11345. 10.1097/md.0000000000011345 PMC6076146 29979411

[73] Kim HG Jin SW Kim YA Khanal T Lee GH Kim SJ. Leptin induces CREB-dependent aromatase activation through COX-2 expression in breast cancer cells. Food Chem Toxicol (2017) 106:232–41. 10.1016/j.fct.2017.05.058 28571770

[74] Linares RL Benitez JGS Reynoso MO Romero CG Sandoval-Cabrera A. Modulation of the leptin receptors expression in breast cancer cell lines exposed to leptin and tamoxifen. Sci Rep (2019) 9:19189. 10.1038/s41598-019-55674-x 31844100 PMC7222035

[75] Huang Y Jin Q Su M Ji F Wang N Zhong C. Leptin promotes the migration and invasion of breast cancer cells by upregulating ACAT2. Cell Oncol (Dordrecht) (2017) 40:537–47. 10.1007/s13402-017-0342-8 PMC13001567 28770546

[76] He JY Wei XH Li SJ Liu Y Hu HL Li ZZ. Adipocyte-derived IL-6 and leptin promote breast Cancer metastasis via upregulation of Lysyl Hydroxylase-2 expression. Cell Commun Signal (2018) 16:100. 10.1186/s12964-018-0309-z 30563531 PMC6299564

[77] Li K Wei L Huang Y Wu Y Su M Pang X. Leptin promotes breast cancer cell migration and invasion via IL-18 expression and secretion. Int J Oncol (2016) 48:2479–87. 10.3892/ijo.2016.3483 27082857

[78] Maccio A Madeddu C Gramignano G Mulas C Floris C Massa D. Correlation of body mass index and leptin with tumor size and stage of disease in hormone-dependent postmenopausal breast cancer: preliminary results and therapeutic implications. J Mol Med (Berl) (2010) 88:677–86. 10.1007/s00109-010-0611-8 20339829

[79] Barone I Giordano C Bonofiglio D Ando S Catalano S. The weight of obesity in breast cancer progression and metastasis: Clinical and molecular perspectives. Semin Cancer Biol (2020) 60:274–84. 10.1016/j.semcancer.2019.09.001 31491560

[80] Wang L Tang C Cao H Li K Pang X Zhong L. Activation of IL-8 via PI3K/Akt-dependent pathway is involved in leptin-mediated epithelial-mesenchymal transition in human breast cancer cells. Cancer Biol Ther (2015) 16:1220–30. 10.1080/15384047.2015.1056409 PMC4622725 26121010

[81] Wei L Li K Pang X Guo B Su M Huang Y. Leptin promotes epithelial-mesenchymal transition of breast cancer via the upregulation of pyruvate kinase M2. J Exp Clin Cancer Res (2016) 35:166. 10.1186/s13046-016-0446-4 27769315 PMC5073421

[82] Sabol R Villela V Denys A Freeman B Hartono A Wise R. Obesity-Altered Adipose Stem Cells Promote Radiation Resistance of Estrogen Receptor Positive Breast Cancer through Paracrine Signaling. Int J Mol Sci (2020) 21:8. 10.3390/ijms21082722 PMC7216284 32326381

[83] Gonzalez RR Watters A Xu Y Singh UP Mann DR Rueda BR. Leptin-signaling inhibition results in efficient anti-tumor activity in estrogen receptor positive or negative breast cancer. Breast Cancer Res (2009) 11:1–12. 10.1186/bcr2321 PMC2716504 19531256

[84] Lipsey C Harbuzariu A Robey R Huff L Gottesman M Gonzalez-Perez R. Leptin Signaling Affects Survival and Chemoresistance of Estrogen Receptor Negative Breast Cancer. Int J Mol Sci (2020) 21:11. 10.3390/ijms21113794 PMC7311967 32471192

[85] Patel DA Srinivasan SR Xu JH Chen W Berenson GS. Adiponectin and its correlates of cardiovascular risk in young adults: the Bogalusa Heart Study. Metabolism (2006) 55:1551–7. 10.1016/j.metabol.2006.06.028 17046560

[86] Ryo M Nakamura T Kihara S Kumada M Shibazaki S Takahashi M. Adiponectin as a biomarker of the metabolic syndrome. Circ J (2004) 68:975–81. 10.1253/circj.68.975 15502375

[87] Mojiminiyi OA Abdella NA Al Arouj M Ben Nakhi A. Adiponectin, insulin resistance and clinical expression of the metabolic syndrome in patients with Type 2 diabetes. Int J Obes (2007) 31:213–20. 10.1038/sj.ijo.0803355 16755284

[88] Santaniemi M Kesaniemi YA Ukkola O. Low plasma adiponectin concentration is an indicator of the metabolic syndrome. Eur J Endocrinol (2006) 155:745–50. 10.1530/eje.1.02287 17062891

[89] Yamauchi T Kamon J Waki H Terauchi Y Kubota N Hara K. The fat-derived hormone adiponectin reverses insulin resistance associated with both lipoatrophy and obesity. Nat Med (2001) 7:941–6. 10.1038/90984 11479627

[90] Khan S Shukla S Sinha S Meeran S. Role of adipokines and cytokines in obesity-associated breast cancer: therapeutic targets. Cytokine Growth Factor Rev (2013) 24:503–13. 10.1016/j.cytogfr.2013.10.001 24210902

[91] Kadowaki T Yamauchi T. Adiponectin and adiponectin receptors. Endocr Rev (2005) 26:439–51. 10.1210/er.2005-0005 15897298

[92] Lam J Chow K Xu A Lam K Liu J Wong N. Adiponectin haploinsufficiency promotes mammary tumor development in MMTV-PyVT mice by modulation of phosphatase and tensin homolog activities. PloS One (2009) 4:e4968. 10.1371/journal.pone.0004968 19319191 PMC2656613

[93] Bråkenhielm E Veitonmäki N Cao R Kihara S Matsuzawa Y Zhivotovsky B. Adiponectin-induced antiangiogenesis and antitumor activity involve caspase-mediated endothelial cell apoptosis. Proc Natl Acad Sci USA (2004) 101:2476–81. 10.1073/pnas.0308671100 PMC356975 14983034

[94] Barb D Pazaitou-Panayiotou K Mantzoros CS. Adiponectin: a link between obesity and cancer. Expert Opin Investig Drugs (2006) 15:917–31. 10.1517/13543784.15.8.917 16859394

[95] Chen H Montagnani M Funahashi T Shimomura I Quon M. Adiponectin stimulates production of nitric oxide in vascular endothelial cells. J Biol Chem (2003) 278:45021–6. 10.1074/jbc.M307878200 12944390

[96] Dubois V Delort L Billard H Vasson MP Caldefie-Chezet F. Breast cancer and obesity: in vitro interferences between adipokines and proangiogenic features and/or antitumor therapies? PloS One (2013) 8:e58541. 10.1371/journal.pone.0058541 23554900 PMC3598910

[97] Mauro L Pellegrino M Giordano F Ricchio E Rizza P De Amicis F. Estrogen receptor-alpha drives adiponectin effects on cyclin D1 expression in breast cancer cells. FASEB J (2015) 29:2150–60. 10.1096/fj.14-262808 25657113

[98] Oh SW Park CY Lee ES Yoon YS Lee ES Park SS. Adipokines, insulin resistance, metabolic syndrome, and breast cancer recurrence: a cohort study. Breast Cancer Res (2011) 13:1–10. 10.1186/bcr2856 PMC3219197 21450081

[99] Pfeiler GH Buechler C Neumeier M Schaffler A Schmitz G Ortmann O. Adiponectin effects on human breast cancer cells are dependent on 17-β estradiol. Oncol Rep (2008) 19:787–93. 10.3892/or.19.3.787 18288417

[100] Landskroner-Eiger S Qian B Muise ES Nawrocki AR Berger JP Fine EJ. Proangiogenic Contribution of Adiponectin toward Mammary Tumor Growth In vivo. Clin Cancer Res (2009) 15:3265–76. 10.1158/1078-0432.CCR-08-2649 PMC3237387 19447867

[101] Chen X Wang Y. Adiponectin and breast cancer. Med Oncol (2011) 28:1288–95. 10.1007/s12032-010-9617-x 20625941

[102] Ciccarese F Zulato E Indraccolo S. LKB1/AMPK Pathway and Drug Response in Cancer: A Therapeutic Perspective. Oxid Med Cell Longev (2019) 2019:8730816. 10.1155/2019/8730816 31781355 PMC6874879

[103] Taliaferro-Smith L Nagalingam A Zhong D Zhou W Saxena NK Sharma D. LKB1 is required for adiponectin-mediated modulation of AMPK-S6K axis and inhibition of migration and invasion of breast cancer cells. Oncogene (2009) 28:2621–33. 10.1038/onc.2009.129 PMC2945727 19483724

[104] Chung SJ Nagaraju GP Nagalingam A Muniraj N Kuppusamy P Walker A. ADIPOQ/adiponectin induces cytotoxic autophagy in breast cancer cells through STK11/LKB1-mediated activation of the AMPK-ULK1 axis. Autophagy (2017) 13:1386–403. 10.1080/15548627.2017.1332565 PMC5584870 28696138

[105] Falk Libby E Liu J Li YI Lewis MJ Demark-Wahnefried W Hurst DR. Globular adiponectin enhances invasion in human breast cancer cells. Oncol Lett (2016) 11:633–41. 10.3892/ol.2015.3965 PMC4726973 26870258

[106] Libby E Frost A Demark-Wahnefried W Hurst D. Linking adiponectin and autophagy in the regulation of breast cancer metastasis. J Mol Med (Berl) (2014) 92:1015–23. 10.1007/s00109-014-1179-5 PMC4197061 24903246

[107] Mauro L Pellegrino M De Amicis F Ricchio E Giordano F Rizza P. Evidences that estrogen receptor α interferes with adiponectin effects on breast cancer cell growth. Cell Cycle (2014) 13:553–64. 10.4161/cc.27455 24335340

[108] Chung SJ Nagaraju GP Nagalingam A Muniraj N Kuppusamy P Walker A. Abstract 3319: Elevating adipokine adiponectin level can induce cytotoxic autophagy in breast cancer cells and potentiate the efficacy of chemotherapeutic regimens: preclinical studies. Cancer Res (2017) 77:3319–9. 10.1158/1538-7445.AM2017-3319

[109] Yunusova NV Kondakova IV Kolomiets LA Afanas’ev SG Chernyshova AL Kudryavtsev IV. Molecular targets for the therapy of cancer associated with metabolic syndrome (transcription and growth factors). Asia Pac J Clin Oncol (2018) 14:134–40. 10.1111/ajco.12780 29115033

[110] Mcternan PG Anwar A Eggo MC Barnett AH Stewart PM Kumar S. Gender differences in the regulation of P450 aromatase expression and activity in human adipose tissue. Int J Obes (2000) 24:875–81. 10.1038/sj.ijo.0801254 10918534

[111] Plymate S Matej L Jones R Friedl K. Inhibition of sex hormone-binding globulin production in the human hepatoma (Hep G2) cell line by insulin and prolactin. J Clin Endocrinol Metab (1988) 67:460–4. 10.1210/jcem-67-3-460 2842359

[112] Xia B Hou L Kang H Chang W Liu Y Zhang Y. NR2F2 plays a major role in insulin-induced epithelial-mesenchymal transition in breast cancer cells. BMC Cancer (2020) 20:626. 10.1186/s12885-020-07107-6 32631390 PMC7336611

[113] Wairagu P Phan A Kim M Han J Kim H Choi J. Insulin priming effect on estradiol-induced breast cancer metabolism and growth. Cancer Biol Ther (2015) 16:484–92. 10.1080/15384047.2015.1016660 PMC4622942 25701261

[114] Fresno Vara JA Casado E de Castro J Cejas P Belda-Iniesta C Gonzalez-Baron M. PI3K/Akt signalling pathway and cancer. Cancer Treat Rev (2004) 30:193–204. 10.1016/j.ctrv.2003.07.007 15023437

[115] Abdul-Rahim H Abu-Rmeileh N Husseini A Holmboe-Ottesen G Jervell J Bjertness E. Obesity and selected co-morbidities in an urban Palestinian population. Int J Obes Relat Metab Disord (2001) 25:1736–40. 10.1038/sj.ijo.0801799 11753598

[116] Mauro L Salerno M Morelli C Boterberg T Bracke ME Surmacz E. Role of the IGF-I receptor in the regulation of cell–cell adhesion: Implications in cancer development and progression. J Cell Physiol (2003) 194:108–16. 10.1002/jcp.10207 12494449

[117] Baserga R. The contradictions of the insulin-like growth factor 1 receptor. Oncogene (2000) 19:5574–81. 10.1038/sj.onc.1203854 11114737

[118] Roith DL. Regulation of proliferation and apoptosis by the insulin-like growth factor I receptor. Growth Horm IGF Res (2000) 10 Suppl A:S12–3. 10.1016/s1096-6374(00)90005-4 10984274

[119] Surmacz E. Function of the IGF-I Receptor in Breast Cancer. Mammary Gland Biol Neoplasia (2000) 5:95–105. 10.1023/a:1009523501499 10791772

[120] Wulaningsih W Sagoo HK Hamza M Melvin J Holmberg L Garmo H. Serum Calcium and the Risk of Breast Cancer: Findings from the Swedish AMORIS Study and a Meta-Analysis of Prospective Studies. Int J Mol Sci (2016) 17:1487. 10.3390/ijms17091487 PMC5037765 27608013

[121] Deliot N Constantin B. Plasma membrane calcium channels in cancer: Alterations and consequences for cell proliferation and migration. Biochim Biophys Acta (2015) 1848:2512–22. 10.1016/j.bbamem.2015.06.009 26072287

[122] Azimi I Bong AH Poo GXH Armitage K Lok D Roberts-Thomson SJ. Pharmacological inhibition of store-operated calcium entry in MDA-MB-468 basal A breast cancer cells: consequences on calcium signalling, cell migration and proliferation. Cell Mol Life Sci (2018) 75:4525–37. 10.1007/s00018-018-2904-y PMC11105359 30105615

[123] Johnson M Trebak M. ORAI channels in cellular remodeling of cardiorespiratory disease. Cell Calcium (2019) 79:1–10. 10.1016/j.ceca.2019.01.005 30772685 PMC6461505

[124] Derler I Jardin I Romanin C. Molecular mechanisms of STIM/Orai communication. Am J Physiol Cell Physiol (2016) 310:C643–62. 10.1152/ajpcell.00007.2016 PMC4835918 26825122

[125] Li CI Daling JR Tang M-TC Haugen KL Porter PL Malone KE. Use of antihypertensive medications and breast cancer risk among women aged 55 to 74 years. JAMA Intern Med (2013) 173:1629–37. 10.1001/jamainternmed.2013.9071 PMC4112594 23921840

[126] Nelson ER Wardell SE Jasper JS Park S Suchindran S Howe MK. 27-Hydroxycholesterol links hypercholesterolemia and breast cancer pathophysiology. Science (2013) 342:1094–8. 10.1126/science.1241908 PMC3899689 24288332

[127] Vedin LL Lewandowski SA Parini P Gustafsson JA Steffensen KR. The oxysterol receptor LXR inhibits proliferation of human breast cancer cells. Carcinogenesis (2009) 30:575–9. 10.1093/carcin/bgp029 19168586

[128] Binai NA Damert A Carra G Steckelbroeck S Lower J Lower R. Expression of estrogen receptor alpha increases leptin-induced STAT3 activity in breast cancer cells. Int J Cancer (2010) 127:55–66. 10.1002/ijc.25010 19876927

[129] Healy LA Ryan AM Carroll P Ennis D Crowley V Boyle T. Metabolic syndrome, central obesity and insulin resistance are associated with adverse pathological features in postmenopausal breast cancer. Clin Oncol (R Coll Radiol) (2010) 22:281–8. 10.1016/j.clon.2010.02.001 20189371

[130] Stebbing J Sharma A North B Athersuch TJ Zebrowski A Pchejetski D. A metabolic phenotyping approach to understanding relationships between metabolic syndrome and breast tumour responses to chemotherapy. Ann Oncol (2012) 23:860–6. 10.1093/annonc/mdr347 21821546

[131] Litton JK Gonzalez-Angulo AM Warneke CL Buzdar AU Kau SW Bondy M. Relationship between obesity and pathologic response to neoadjuvant chemotherapy among women with operable breast cancer. J Clin Oncol (2008) 26:4072–7. 10.1200/JCO.2007.14.4527 PMC6557586 18757321

[132] Protani M Coory M Martin JH. Effect of obesity on survival of women with breast cancer: systematic review and meta-analysis. Breast Cancer Res Treat (2010) 123:627–35. 10.1007/s10549-010-0990-0 20571870

[133] Hsu MC Lee KT Hsiao WC Wu CH Sun HY Lin IL. The dyslipidemia-associated SNP on the APOA1/ C3/A5 gene cluster predicts post-surgery poor outcome in Taiwanese breast cancer patients: a 10-year follow-up study. BioMed Cent Cancer (2013) 13:330. 10.1186/1471-2407-13-330 PMC3708770 23829168

[134] Griggs J Mangu P Anderson H Balaban E Dignam J Hryniuk W. Appropriate chemotherapy dosing for obese adult patients with cancer: American Society of Clinical Oncology clinical practice guideline. J Clin Oncol (2012) 30:1553–61. 10.1200/jco.2011.39.9436 22473167

[135] Zeng L Zielinska HA Arshad A Shield JP Bahl A Holly JM. Hyperglycaemia-induced chemoresistance in breast cancer cells: role of the estrogen receptor. Endocr Relat Cancer (2016) 23:125–34. 10.1530/ERC-15-0507 26647383

[136] Pfeiler G StöGer H Dubsky P Mlineritsch B Singer C Balic M. Efficacy of tamoxifen ± aminoglutethimide in normal weight and overweight postmenopausal patients with hormone receptor-positive breast cancer: an analysis of 1509 patients of the ABCSG-06 trial. Br J Cancer (2013) 108:1408–14. 10.1038/bjc.2013.114 PMC3629426 23511562

[137] Zhu QL Xu WH Tao MH. Biomarkers of the metabolic syndrome and breast cancer prognosis. Cancers (2010) 2:721–39. 10.3390/cancers2020721 PMC3835101 24281091

[138] Salerno M Sisci D Mauro L Guvakova MA Ando S Surmacz E. Insulin receptor substrate 1 is a target for the pure antiestrogen ICI 182,780 in breast cancer cells. Int J Cancer (1999) 81:299–304. 10.1002/(SICI)1097-0215(19990412)81:23.0.CO;2-8 10188734

[139] D’Esposito V Passaretti F Hammarstedt A Liguoro D Terracciano D Molea G. Adipocyte-released insulin-like growth factor-1 is regulated by glucose and fatty acids and controls breast cancer cell growth in vitro. Diabetologia (2012) 55:2811–22. 10.1007/s00125-012-2629-7 PMC3433668 22798065

[140] Lee A Jo S Lee C Shin HH Kim TH Ahn KJ. Diabetes as a prognostic factor in HER-2 positive breast cancer patients treated with targeted therapy. Breast Cancer (2019) 26:672–80. 10.1007/s12282-019-00967-2 30927244

[141] Park J Sarode VR Euhus D Kittler R Scherer PE. Neuregulin 1-HER axis as a key mediator of hyperglycemic memory effects in breast cancer. Proc Natl Acad Sci USA (2012) 109:21058–63. 10.1073/pnas.1214400109 PMC3529089 23213231

[142] Fang P Tan KS Troxel AB Rengan R Freedman G Lin LL. High body mass index is associated with worse quality of life in breast cancer patients receiving radiotherapy. Breast Cancer Res Treat (2013) 141:125–33. 10.1007/s10549-013-2663-2 23942874

[143] Dieli-Conwright CM Wong L Waliany S Bernstein L Salehian B Mortimer JE. An observational study to examine changes in metabolic syndrome components in patients with breast cancer receiving neoadjuvant or adjuvant chemotherapy. Cancer (2016) 122:2646–53. 10.1002/cncr.30104 PMC4992442 27219902

[144] Bicakli DH Varol U Degirmenci M Tunali D Cakar B Durusoy R. Adjuvant chemotherapy may contribute to an increased risk for metabolic syndrome in patients with breast cancer. J Oncol Pharm Pract (2016) 22:46–53. 10.1177/1078155214551315 25233884

[145] Goodwin P Ennis M Pritchard K McCready D Koo J Sidlofsky S. Adjuvant treatment and onset of menopause predict weight gain after breast cancer diagnosis. J Clin Oncol (1999) 17:120–9. 10.1200/jco.1999.17.1.120 10458225

[146] Cho WK Choi DH Park W Cha H Nam SJ Kim SW. Effect of Body Mass Index on Survival in Breast Cancer Patients According to Subtype, Metabolic Syndrome, and Treatment. Clin Breast Cancer (2018) 18:1141–7. 10.1016/j.clbc.2018.04.010 29753627

[147] Fredslund SO Gravholt CH Laursen BE Jensen AB. Key metabolic parameters change significantly in early breast cancer survivors: an explorative PILOT study. J Transl Med (2019) 17:1–13. 10.1186/s12967-019-1850-2 30935397 PMC6444586

[148] Vigneri P Frasca F Sciacca L Pandini G Vigneri R. Diabetes and cancer. Endocr Relat Cancer (2009) 16:1103–23. 10.1677/ERC-09-0087 19620249

[149] Bordeleau L Lipscombe L Lubinski J Ghadirian P Foulkes WD Neuhausen S. Diabetes and breast cancer among women with BRCA1 and BRCA2 mutations. Cancer (2011) 117:1812–8. 10.1002/cncr.25595 PMC3413077 21509758

[150] Johansson H Gandini S Guerrieri-Gonzaga A Iodice S Ruscica M Bonanni B. Effect of fenretinide and low-dose tamoxifen on insulin sensitivity in premenopausal women at high risk for breast cancer. Cancer Res (2008) 68:9512–8. 10.1158/0008-5472.CAN-08-0553 PMC2599903 19010927

[151] Bundred NJ. The effects of aromatase inhibitors on lipids and thrombosis. Br J Cancer (2005) 93 Suppl 1:S23–7. 10.1038/sj.bjc.6602692 PMC2361692 16100522

[152] Nguyen M Stewart R Banerji M Gordon D Kral J. Relationships between tamoxifen use, liver fat and body fat distribution in women with breast cancer. Int J Obes Relat Metab Disord (2001) 25:296–8. 10.1038/sj.ijo.0801488 11410835

[153] Sun LM Chen HJ Liang JA Li TC Kao CH. Association of tamoxifen use and increased diabetes among Asian women diagnosed with breast cancer. Br J Cancer (2014) 111:1836–42. 10.1038/bjc.2014.488 PMC4453737 25225901

[154] Bell LN Nguyen AT Li L Desta Z Henry NL Hayes DF. Comparison of changes in the lipid profile of postmenopausal women with early stage breast cancer treated with exemestane or letrozole. J Clin Pharmacol (2012) 52:1852–60. 10.1177/0091270011424153 PMC3616612 22174434

[155] Hong N Yoon HG Seo DH Park S Kim SI Sohn JH. Different patterns in the risk of newly developed fatty liver and lipid changes with tamoxifen versus aromatase inhibitors in postmenopausal women with early breast cancer: A propensity score-matched cohort study. Eur J Cancer (2017) 82:103–14. 10.1016/j.ejca.2017.05.002 28651157

[156] Tanaka H Takahashi K Yamaguchi K Kontani K Motoki T Asakura M. Hypertension and Proteinuria as Predictive Factors of Effects of Bevacizumab on Advanced Breast Cancer in Japan. Biol Pharm Bull (2018) 41:644–8. 10.1248/bpb.b17-00605 29607938

[157] Sane DC Anton L Brosnihan KB. Angiogenic growth factors and hypertension. Angiogenesis (2004) 7:193–201. 10.1007/s10456-004-2699-3 15609074

[158] Dawood S Broglio K Buzdar AU Hortobagyi GN Giordano SH. Prognosis of women with metastatic breast cancer by HER2 status and trastuzumab treatment: an institutional-based review. J Clin Oncol (2010) 28:92–8. 10.1200/JCO.2008.19.9844 PMC2799236 19933921

[159] Piccart-Gebhart MJ Procter M Leyland-Jones B Goldhirsch A Untch M Smith I. Trastuzumab after adjuvant chemotherapy in HER2-positive breast cancer. N Engl J Med (2005) 353:1659–72. 10.1056/NEJMoa052306 16236737

[160] Slamon DJ Leyland-Jones B Shak S Fuchs H Paton V Bajamonde A. Use of chemotherapy plus a monoclonal antibody against HER2 for metastatic breast cancer that overexpresses HER2. N Engl J Med (2001) 344:783–92. 10.1056/NEJM200103153441101 11248153

[161] Perez EA Rodeheffer R. Clinical cardiac tolerability of trastuzumab. J Clin Oncol (2004) 22:322–9. 10.1200/JCO.2004.01.120 14722042

[162] Guenancia C Lefebvre A Cardinale D Yu AF Ladoire S Ghiringhelli F. Obesity as a Risk Factor for Anthracyclines and Trastuzumab Cardiotoxicity in Breast Cancer: A Systematic Review and Meta-Analysis. J Clin Oncol (2016) 34:3157–65. 10.1200/JCO.2016.67.4846 PMC5569689 27458291

[163] Gunaldi M Duman BB Afsar CU Paydas S Erkisi M Kara IO. Risk factors for developing cardiotoxicity of trastuzumab in breast cancer patients: An observational single-centre study. J Oncol Pharm Pract (2016) 22:242–7. 10.1177/1078155214567162 25567518

[164] Serrano JM Gonzalez I Del Castillo S Muniz J Morales LJ Moreno F. Diastolic Dysfunction Following Anthracycline-Based Chemotherapy in Breast Cancer Patients: Incidence and Predictors. Oncologist (2015) 20:864–72. 10.1634/theoncologist.2014-0500 PMC4524754 26185196

[165] Rodvold KA Rushing DA Tewksbury DA. Doxorubicin clearance in the obese. J Clin Oncol (1988) 6:1321–7. 10.1200/JCO.1988.6.8.1321 3411343

[166] Mitra MS Donthamsetty S White B Mehendale HM. High fat diet-fed obese rats are highly sensitive to doxorubicin-induced cardiotoxicity. Toxicol Appl Pharmacol (2008) 231:413–22. 10.1016/j.taap.2008.05.006 18674790

[167] Maruyama S Shibata R Ohashi K Ohashi T Daida H Walsh K. Adiponectin Ameliorates Doxorubicin-induced Cardiotoxicity through Akt Protein-dependent Mechanism. J Biol Chem (2011) 286:32790–800. 10.1074/jbc.M111.245985 PMC3173230 21784858

[168] Muniz J Kidwell KM Henry NL. Associations between metabolic syndrome, breast cancer recurrence, and the 21-gene recurrence score assay. Breast Cancer Res Treat (2016) 157:597–603. 10.1007/s10549-016-3846-4 27271766 PMC5095927

[169] Goodwin PJ Ennis M Pritchard KI Trudeau ME Koo J Madarnas Y. Fasting Insulin and Outcome in Early-Stage Breast Cancer: Results of a Prospective Cohort Study. J Clin Oncol (2002) 20:42–51. 10.1200/JCO.2002.20.1.42 11773152

[170] Emaus A Veierd MB Tretli S Finstad SE Selmer R Furberg AS. Metabolic profile, physical activity, and mortality in breast cancer patients. Breast Cancer Res Treat (2010) 121:651–60. 10.1007/s10549-009-0603-y 19882245

[171] Minicozzi P Berrino F Sebastiani F Falcini F Vattiato R Cioccoloni F. High fasting blood glucose and obesity significantly and independently increase risk of breast cancer death in hormone receptor-positive disease. Eur J Cancer (2013) 49:3881–8. 10.1016/j.ejca.2013.08.004 24011933

[172] Bahl M Ennis M Tannock IF Hux JE Pritchard KI Koo J. Serum Lipids and Outcome of Early-stage Breast Cancer: Results of a Prospective Cohort Study. Breast Cancer Res Treat (2005) 94:135–44. 10.1007/s10549-005-6654-9 16261412

[173] Jung SM Kang D Guallar E Yu J Lee JE Kim SW. Impact of Serum Lipid on Breast Cancer Recurrence. J Clin Med (2020) 9:2846. 10.3390/jcm9092846 PMC7564113 32887448

[174] Özdemir BH Akcali Z Haberal M. Hypercholesterolemia Impairs Angiogenesis in Patients with Breast Carcinoma and, Therefore, Lowers the Risk of Metastases. Am J Clin Pathol (2004) 122:696–703. 10.1309/hw2myb5tvf4am0y4 15491965

[175] Braithwaite D Tammemagi CM Moore DH Ozanne EM Hiatt RA Belkora J. Hypertension is an independent predictor of survival disparity between African-American and white breast cancer patients. Int J Cancer (2009) 124:1213–9. 10.1002/ijc.24054 19058216

[176] Tominaga S Kuroishi T. Epidemiology and prevention of Breast Cancer in the 21st century. Breast Cancer (1999) 6:283–8. 10.1007/BF02966440 11091730

[177] Ng EH Gao F Ji CY Ho GH Soo KC. Risk factors for breast carcinoma in Singaporean Chinese women: the role of central obesity. Cancer (1997) 80:725–31. 10.1002/(sici)1097-0142(19970815)80:4<725::aid-cncr11>3.0.co;2-v 9264356

[178] Jiralerspong S Kim ES Dong W Feng L Hortobagyi GN Giordano SH. Obesity, diabetes, and survival outcomes in a large cohort of early-stage breast cancer patients. Ann Oncol (2013) 24:2506–14. 10.1093/annonc/mdt224 PMC3784334 23793035

[179] Peairs K Barone B Snyder C Yeh H Stein K Derr R. Diabetes mellitus and breast cancer outcomes: a systematic review and meta-analysis. J Clin Oncol (2011) 29:40–6. 10.1200/jco.2009.27.3011 PMC3055858 21115865

[180] Zhao X Ren G. Diabetes mellitus and prognosis in women with breast cancer: A systematic review and meta-analysis. Med (Baltimore) (2016) 95:1–7. 10.1097/md.0000000000005602 PMC5266055 27930583

[181] Bozcuk H Uslu G Samur M YiLdiZ M Ozben T OZdogan M. Tumour necrosis factor-alpha, interleukin-6, and fasting serum insulin correlate with clinical outcome in metastatic breast cancer patients treated with chemotherapy. Cytokine (2004) 27:58–65. 10.1016/j.cyto.2004.04.002 15242694

[182] Pasanisi P Berrino F Petris MD Venturelli E Mastroianni A Panico S. Metabolic syndrome as a prognostic factor for breast cancer recurrences. Int J Cancer (2010) 119:236–8. 10.1002/ijc.21812 16450399

[183] Jiralerspong S Palla SL Giordano SH Meric-Bernstam F Liedtke C Barnett CM. Metformin and pathologic complete responses to neoadjuvant chemotherapy in diabetic patients with breast cancer. J Clin Oncol (2009) 27:3297–302. 10.1200/JCO.2009.19.6410 PMC2736070 19487376

[184] Giles ED Jindal S Wellberg EA Schedin T Anderson SM Thor AD. Metformin inhibits stromal aromatase expression and tumor progression in a rodent model of postmenopausal breast cancer. Breast Cancer Res (2018) 20:50. 10.1186/s13058-018-0974-2 29898754 PMC6000949

[185] Li YR Ro V Steel L Carrigan E Nguyen J Williams A. Impact of long-term lipid-lowering therapy on clinical outcomes in breast cancer. Breast Cancer Res Treat (2019) 176:669–77. 10.1007/s10549-019-05267-z 31087198

[186] Borgquist S Giobbie-Hurder A Ahern T Garber J Colleoni M Láng I. Cholesterol, Cholesterol-Lowering Medication Use, and Breast Cancer Outcome in the BIG 1-98 Study. J Clin Oncol (2017) 35:1179–88. 10.1200/jco.2016.70.3116 28380313

[187] Ibbotson SH Davies JA Grant PJ. Statins Can Inhibit Proliferation of Human Breast Cancer Cells in Vitro. Exp Clin Endocrinol Diabetes (2003) 111:47–8. 10.1055/s-2003-37501 12605351

[188] Ghosh-Choudhury N Mandal CC Ghosh-Choudhury N Ghosh Choudhury G. Simvastatin induces derepression of PTEN expression via NFkappaB to inhibit breast cancer cell growth. Cell Signal (2010) 22:749–58. 10.1016/j.cellsig.2009.12.010 PMC2826504 20060890

[189] Campbell MJ. Breast Cancer Growth Prevention by Statins. Cancer Res (2006) 66:8707–14. 10.1158/0008-5472.CAN-05-4061 16951186

[190] Mueck AO Seeger H Wallwiener D. Effect of statins combined with estradiol on the proliferation of human receptor-positive and receptor-negative breast cancer cells. Menopaus (2003) 10:332–6. 10.1097/01.GME.0000055485.06076.00 12851516

[191] Giacosa A Barale R Bavaresco L Gatenby P Gerbi V Janssens J. Cancer prevention in Europe: the Mediterranean diet as a protective choice. Eur J Cancer Prev (2013) 22:90–5. 10.1097/CEJ.0b013e328354d2d7 22644232

[192] Trichopoulou A Bamia C Lagiou P Trichopoulos D. Conformity to traditional Mediterranean diet and breast cancer risk in the Greek EPIC (European Prospective Investigation into Cancer and Nutrition) cohort. Am J Clin Nutr (2010) 92:620–5. 10.3945/ajcn.2010.29619 20631204

[193] Vanessa C Mathilde T Agnès F Touillaud MS Lionel L Françoise CC. Postmenopausal Breast Cancer Risk and Dietary Patterns in the E3N-EPIC Prospective Cohort Study. Am J Epidemiol (2009) 170:1257–67. 10.1093/aje/kwp257 19828509

[194] Willett WC Sacks F Trichopoulou A Drescher G Ferro-Luzzi A Helsing E. Mediterranean diet pyramid: A cultural model for healthy eating. Am J Clin Nutr (1995) 61:1402–6. 10.1093/ajcn/61.6.1402S 7754995

[195] Berrino F Villarini A De Petris M Raimondi M Pasanisi P. Adjuvant diet to improve hormonal and metabolic factors affecting breast cancer prognosis. Ann N Y Acad Sci (2006) 1089:110–8. 10.1196/annals.1386.023 17261760

[196] Mirabelli M Chiefari E Arcidiacono B Corigliano DM Brunetti FS Maggisano V. Mediterranean Diet Nutrients to Turn the Tide against Insulin Resistance and Related Diseases. Nutrients (2020) 12:1066. 10.3390/nu12041066 PMC7230471 32290535

[197] Mengxi D Liu SH Cara M Fung TT. Associations between Diet Quality Scores and Risk of Postmenopausal Estrogen Receptor-Negative Breast Cancer: A Systematic Review. J Nutr (2018) 148:100–8. 10.1093/jn/nxx015 29378048

